# Supplementary material for: Cohort Profile: CArdiovascular Risk in patients with DIAbetes in NAvarra (CARDIANA cohort)
Source: BMJ Open. 2023 Jan 20;13(1):e066052. doi: 10.1136/bmjopen-2022-066052 (PMC9872484; doi:10.1136/bmjopen-2022-066052)
Supplement: Supplementary data [file bmjopen-2022-066052supp001.pdf]

Supplementary Table S1: Original Data Sources used for the creation of the T1D and T2D cohorts[Ω]

ATENEA –The Primary Care Electronic Medical Record System of Navarra
What contains: out-hospital data. Includes demographic information, visits to primary care services (data and type), health problems, life style, detailed clinical data, laboratory results and drug prescription data.<br>*Code system used:* ICPC-2 International Classification of Primary Care, version 2<br>*Implemented:* in 2003, with a 100% of use in 2008
LAKORA – Population Information System (within the Health system)
What contains: Basic information on coverage, insurance modality, pharmaceutical copayment status, primary healthcare district, country of origin and other administrative data.<br>*Code system used*: free text<br>*Implemented: in 2003,* with a 100% of use in 2008
MORTALITY Registry
What contains: Date and cause of death<br>*Code system used*: International Classification of Diseases ICD-10-ES
HIS-LEIRE (with the Minimum Basic Data Set, MBDS)
What contains: It contains the MBDS, with socio-demographic and clinical information on all hospital discharges and major ambulatory surgery in the hospitals of Navarra, including partnership hospitals. It also contains information about visits to specialist or to day hospitals<br>*Code system used*: International Classification of Diseases; ICD9CM until December, 2015 and ICD-10-ES from January, 2016 on.<br>*Implemented*: The MBDS was compulsory in Spain since 1992. HIS-LEIRE had 100% of use in 2003
HCI- Clinical data from specialized care
What contains: comprehensive information of specialized care, outpatient consultation, lab test, images, visits and other data from specialized care.<br>*Code system used*: free text<br>*Implemented*: In 2001, with 100% of use in 2008
T1D registry
What contains: demographic and clinical information of all patients with T1D diabetes to estimate incidence, prevalence, morbidity and mortality of T1D patients<br>*Implemented*: in 2014, legally approved by formal order 37/2014
Population registry
What contains: At individual level: study level, nationality, labor force status (employed, unemployed, pensioner); at area-level (census tract): average income (2013), % unemployed, %immigration, % people without studies<br>*Code system used*: For study level: Normalized Classification of Education (CNE) used for census in the INE (National Institute of Statistics)[£]
LAMIA
What contains: e-prescription paper-free system with both prescriptions and dispensations, connected to all community pharmacies in the region<br>*Code system used*: Anatomical Therapeutic Chemical (ATC) code<br>*Implemented*: In 2013, with 100% of use in 2014. Before 2014, information on prescriptions was obtained from ATENEA.

[Ω] For future updates of the cohort, the Results Analysis Database of Navarre (BARDENA) will be used

[£] https://idapadron.ine.es/repositorio/legislacion/Anexo%20II.pdf

Supplementary Table S2: Data information available in the T1D and T2D cohorts, by type of information and data source

Data source	Variables
**Socioeconomic data**	
Population Registry	Date of birth, country of origin, study level, marital status, working status and mean income of the assigned basic health zone
LAKORA-TIS	Pharmaceutical co-payment category
ATENEA-TIS	Date of birth, date of inclusion/withdrawal in the Navarra health system, general practitioner identification
**Previous diagnosis and past history of diseases**	
ATENEA	Weighted GMA, Past-history of myocardial infarction, congestive heart failure, cerebrovascular disease, peripheral vascular disease, retinopathy, neuropathy, nephropathy, foot lesions, dementia, chronic pulmonary disease, rheumatic disease, peptic ulcer disease, hemiplegia or paraplegia, neoplasm, liver disease, AIDS/HIV. Past history of CVD events coded in one of the following ICPC-2 codes: K74, K75, K76, K77, K89, K90, K91
**Personal cardiovascular risk factors, laboratory test and lifestyle behavior**	
ATENEA	Sex, age, physical activity, alcohol consumption, smoking status, self-management of pharmacological treatment, time since diagnosis, office, and laboratory test parameters (and dates): weight, height, systolic blood pressure (SBP), diastolic blood pressure (DBP), Total Cholesterol, LDL, HDL, triglycerides, creatinine, haemoglobin, glycated haemoglobin, fasting glucose, glomerular filtration rate (GFR), urine albumin to creatinine ratio.
The HCI database	Data collected by specialist care units: office and laboratory test parameters and procedures (date and value)
**Use of health services**	
HIS-LEIRE	Hospital or emergency room admissions, diagnostics, procedures and dates of admission and discharge. Number of consultations to specialist by medical specialty
ATENEA	Number of yearly telephonic/physical consultation, office/home-based consultation and type of service provided (nurse/ medical/social worker/Emergency room)
**Drug treatment use**	
LAMIA	Treatment ATC code, date of prescription and date of dispensation
ATENEA	Treatment ATC code and date of prescription (before 2014)
**Diabetes classification**	
T1D registry	Type 1 diabetes confirmation and onset date
**Outcomes**	
Mortality registry	Cause of death and date ICD-10-ES
HIS-LEIRE/MBDS	Minimum Basic Data Set (MBDS): ICD-9CM until December 31st, 2015 and ICD-10-ES from January 1st, 2016.

Supplementary Table S3: ICD-9 and ICD-10 codes for the definition of the outcome

**Fatal CVD event**		
Endpoints included	*ICD10-codes*	*ICD9-codes*
Hypertensive disease	I10-16	401 – 405
Ischemic heart disease	I20-25	410 - 414
Arrhythmias, heart failure	I46-52	426 - 429
Cerebrovascular disease	I60-69	430 - 438
Atherosclerosis/AAA	I70-73	440 - 443
Sudden death and death within 24h of symptom onset	R96.0-96.1	798.1 , 798.2
Excluded from the above endpoint:		
Myocarditis, unspecified	I51.4	426.7
Subarachnoid haemorrhage	I60	429
Subdural haemorrhage	I62	430
Cerebral aneurysm	I67.1	432.1
Cerebral arteritis	I68.2	437.3
Moyamoya	I67.5	437.4
Non-fatal CVD event		
Endpoints included		
Non-fatal myocardial infarction	I21-I23	410
Non-fatal stroke	I60-69	430-438
Excluded from the non-fatal stroke endpoint:		
Subarachnoid hemorrhage	I60	429
Subdural hemorrhage	I62	430
Cerebral aneurysm	I67.1	432.1
Cerebral arteritis	I68.2	437.3
Moyamoya	I67.5	437.4

Cardiovascular Disease (CVD) event defined with Codes considered in SCORE2 [16]
Only primary codes are considered
