# Supplementary material for: Cohort Profile: CArdiovascular Risk in patients with DIAbetes in NAvarra (CARDIANA cohort)
Source: BMJ Open. 2023 Jan 20;13(1):e066052. doi: 10.1136/bmjopen-2022-066052 (PMC9872484; doi:10.1136/bmjopen-2022-066052)
Supplement: Author's manuscript [file bmjopen-2022-066052.draft_revisions.pdf]

BMJ Open is committed to open peer review. As part of this commitment we make the peer review history of every article we publish publicly available.

When an article is published we post the peer reviewers' comments and the authors' responses online. We also post the versions of the paper that were used during peer review. These are the versions that the peer review comments apply to.

The versions of the paper that follow are the versions that were submitted during the peer review process. They are not the versions of record or the final published versions. They should not be cited or distributed as the published version of this manuscript.

BMJ Open is an open access journal and the full, final, typeset and author-corrected version of record of the manuscript is available on our site with no access controls, subscription charges or pay-per-view fees (http://bmjopen.bmj.com).

If you have any questions on BMJ Open's open peer review process please email info.bmjopen@bmj.com

BMJ Open

Cohort Profile: CARDIANA cohort: CArdiovascular Risk in patients with DIAbetes in Navarra

Journal:	*BMJ Open*
Manuscript ID	bmjopen-2022-066052
Article Type:	Cohort profile
Date Submitted by the Author:	28-Jun-2022
Complete List of Authors:	Tamayo, Ibai; Navarrabiomed, IdISNA; Red de Investigación en Servicios de Salud en Enfermedades Crónicas, Red de Investigación en Cronicidad, Atención Primaria y Promoción de la Salud<br>Librero-Lopez, Julian ; Navarrabiomed, IdiSNA; Red de Investigacion en Servicios de Salud en Enfermedades Cronicas, Red de Investigación en Cronicidad, Atención Primaria y Servicios de Salud<br>Galbete, Arkaitz; Universidad Pública de Navarra, Estadística; Navarrabiomed, IdISNA<br>Cambra, Koldo; Gobierno Vasco, Departamento de Sanidad; Red de Investigación en Servicios de Salud en Enfermedades Crónicas, Red de Investigación en Cronicidad, Atención Primaria y Promoción de la Salud<br>Enguita, Mónica; Navarrabiomed, IdiSNA; Red de Investigación en Servicios de Salud en Enfermedades Crónicas, Red de Investigación en Cronicidad, Atención Primaria y Promoción de la Salud<br>Forga, Luis; Servicio Navarro de Salud - Osasunbidea, Servicio de Endocrinología y Nutrición - HUN; Red de Investigación en Servicios de Salud en Enfermedades Crónicas, Red de Investigación en Cronicidad, Atención Primaria y Promoción de la Salud<br>Goñi, José; Servicio Navarro de Salud - Osasunbidea, Servicio de Endocrinología y Nutrición - HUN; Red de Investigación en Servicios de Salud en Enfermedades Crónicas, Red de Investigación en Cronicidad, Atención Primaria y Promoción de la Salud<br>Lecea, Oscar; Servicio Navarro de Salud - Osasunbidea, Atención Primaria; Red de Investigación en Servicios de Salud en Enfermedades Crónicas, Red de Investigación en Cronicidad, Atención Primaria y Promoción de la Salud<br>Gorricho, Javier; Servicio Navarro de Salud - Osasunbidea, Servicio de Evaluación y Difusión de resultados en Salud; Red de Investigacion en Servicios de Salud en Enfermedades Cronicas, Red de Investigación en Cronicidad, Atención Primaria y Servicios Sanitarios<br>Olazarán, Álvaro; Departamento de Universidad, Innovación y Transformación, Servicio Tecnologías de la Salud<br>Arnedo, Laura; Instituto de Estadística de Navarra<br>Moreno-Iribas , Conchi; Instituto de Salud Pública y Laboral de Navarra; Red de Investigacion en Servicios de Salud en Enfermedades Cronicas, Red de Investigación en Cronicidad, Atención Primaria y Promoción de la Salud<br>Lafita, Javier; Servicio Navarro de Salud - Osasunbidea, Servicio de Efectividad y Seguridad Asistencial ; Red de Investigación en Servicios de Salud en Enfermedades Crónicas, Red de Investigación en Cronicidad, Atención Primaria y Promoción de la Salud

	Ibañez, Berta; Navarrabiomed, IdiSNA; Red de Investigacion en Servicios de Salud en Enfermedades Cronicas, Red de Investigación en Cronicidad, Atención Primaria y Promoción de la Salud

Keywords:	General diabetes < DIABETES & ENDOCRINOLOGY, Cardiac Epidemiology < CARDIOLOGY, Risk management < HEALTH SERVICES ADMINISTRATION & MANAGEMENT

SCHOLARONE™ Manuscripts

I, the Submitting Author has the right to grant and does grant on behalf of all authors of the Work (as defined in the below author licence), an exclusive licence and/or a non-exclusive licence for contributions from authors who are: i) UK Crown employees; ii) where BMJ has agreed a CC-BY licence shall apply, and/or iii) in accordance with the terms applicable for US Federal Government officers or employees acting as part of their official duties; on a worldwide, perpetual, irrevocable, royalty-free basis to BMJ Publishing Group Ltd ("BMJ") its licensees and where the relevant Journal is co-owned by BMJ to the co-owners of the Journal, to publish the Work in this journal and any other BMJ products and to exploit all rights, as set out in our licence.

The Submitting Author accepts and understands that any supply made under these terms is made by BMJ to the Submitting Author unless you are acting as an employee on behalf of your employer or a postgraduate student of an affiliated institution which is paying any applicable article publishing charge ("APC") for Open Access articles. Where the Submitting Author wishes to make the Work available on an Open Access basis (and intends to pay the relevant APC), the terms of reuse of such Open Access shall be governed by a Creative Commons licence – details of these licences and which Creative Commons licence will apply to this Work are set out in our licence referred to above.

Other than as permitted in any relevant BMJ Author's Self Archiving Policies, I confirm this Work has not been accepted for publication elsewhere, is not being considered for publication elsewhere and does not duplicate material already published. I confirm all authors consent to publication of this Work and authorise the granting of this licence.

For peer review only

TITLE PAGE

TITLE: Cohort Profile: CARDIANA cohort: CArdiovascular Risk in patients with DIAbetes in Navarra

Ibai Tamayo[a,b] , Julián Librero[a,b], Arkaitz Galbete[a,c], Koldo Cambra[b,d], Mónica Enguita[a,b], Luis Forga[b,e], Mª José Goñi[b,e], Oscar Lecea[b,f], Javier Gorricho[b,g], Álvaro Olazarán[h], Laura Arnedo[i], Conchi Moreno-Iribas[b,j], Javier Lafita[b,k] and Berta Ibáñez-Beroiz *[a,b]

a. Unidad de Metodología. Navarrabiomed-Hospital Universitario de Navarra-Universidad Pública de Navarra. IdiSNA. Pamplona. Spain

b. Red de Investigación en Servicios Sanitarios y Enfermedades Crónicas (REDISSEC) y Red de Investigación en Cronicidad, Atención Primaria y Promoción de la Salud (RICAPPS). Pamplona. Spain

c. Departamento de Estadística. Universidad Pública de Navarra (UPNA). Pamplona. Spain

d. Dirección de Salud Pública y Adicciones. Departamento de Sanidad. Gobierno Vasco. Vitoria. Spain

e. Servicio de Endocrinología y Nutrición, Hospital Universitario de Navarra, Servicio Navarro de Salud (SNS-O). Pamplona. Spain

f. Atención Primaria, Servicio Navarro de Salud (SNS-O). Pamplona. Spain

g. Servicio de Evaluación y Difusión de resultados en Salud, Servicio Navarro de Salud (SNS-O). Pamplona. Spain

h. Servicio Tecnologías de la Salud, Dpto de Universidad, Innovación y Transformación. Pamplona. Spain

i. Instituto de Estadística de Navarra (NASTAT). Pamplona. Spain

j. Instituto de Salud Pública y Laboral de Navarra (ISPLN). Pamplona. Spain

k. Servicio de Efectividad y Seguridad Asistencial (SNS-O). Pamplona. Spain

For peer review only

For peer review only - http://bmjopen.bmj.com/site/about/guidelines.xhtml

ibai.tamayo.rodriguez@navarra.es

julian.librero.lopez@navarra.es

arkaitz.galbete@unavarra.es

k-cambra@euskadi.eus

monica.enguita.german@navarra.es

lluis.forga.llenas@navarra.es

mj.goni.iriarte@navarra.es

oscar.lecea.juarez@navarra.es

javier.gorricho.mendivil@navarra.es

alvaro.olazaran.santesteban@navarra.es

laura.arnedo.ajona@navarra.es

mc.moreno.iribas@navarra.es

javier.lafita.tejedor@navarra.es

CORRESPONDING AUTHOR: Berta Ibáñez-Beroiz berta.ibanez.beroiz@navarra.es

Navarrabiomed-Complejo Hospitalario de Navarra-Universidad Pública de Navarra

C/ Irunlarrea s/n. Recinto CHN. 31008. Pamplona. Spain Tfno: 0034848428393;

Word count: 2871

For peer review only

For peer review only - http://bmjopen.bmj.com/site/about/guidelines.xhtml

Abstract

Purpose: The CARDIANA cohort was established to unravel the effects of sociodemographic and clinical variables on the risk of cardiovascular events in patients with type 1 (T1D) or type 2(T2D) diabetes, with a special focus on socioeconomic factors, and to validate and develop cardiovascular risk models for these patients.

Participants: The CARDIANA cohort included all patients with type 1 (T1D) and type 2 (T2D) diabetes registered in the Public Health Service of Navarra with prevalent disease on January 1, 2012. It consisted of 1067 T1D patients (ages 2-88 years) and 33842 T2D patients (ages 20-105 years), whose data were retrospectively extracted from the Health and Administrative System Databases.

Findings to date: The follow-up period for Wave 1 was from January 1, 2012 to December 31, 2016. During these five years, 17 patients (1.6%; 95% CI [1.0, 2.5]) in the T1D cohort developed a CVD event, whereas for the T2D cohort, 2882 (8.5%; 95% CI [8.2, 8.8]) had an event. Some individual factors, such as being physically active, have been found associated with CVD event occurrence in these patients.

Future plans: The CARDIANA cohort is being used to externally validate cardiovascular predictive models. A second Wave will be conducted during autumn of 2022, to extend the follow-up other five years, from January 1, 2016 to December 31, 2021.

For peer review only

For peer review only - http://bmjopen.bmj.com/site/about/guidelines.xhtml

Strengths and limitations

- The CARDIANA cohorts integrate exhaustive clinical, socioeconomic and behavioral information from all available administrative and clinical data sources in patients with T1D and T2D diabetes.
- The data have been subject to quality control procedures before and after database integration.
- The presence of possible bias resulting from the use of existing electronic clinical records require to be accounted for.
- Some variables may be underreported and electronic prescriptions were only fully implemented in 2014.

For peer review only

For peer review only - http://bmjopen.bmj.com/site/about/guidelines.xhtml

Introduction

Diabetes mellitus is a common metabolic disorder (537 million people worldwide in 2021)[1] that is characterized by chronic hyperglycemia and an imbalance in lipid, carbohydrate, and protein metabolism.[2] The impact of diabetes on the population is huge, being a major driver of premature deaths and catastrophic health care expenditure. According to current estimations, 11.5% of the total health care spending and 12.2% of global all-cause deaths in adults aged 20-79 years are attributable to diabetes.[1,3] Despite governments agreeing to halt the increase in diabetes and obesity by 2025, the probability of dying prematurely from diabetes increased by 5% between 2000 and 2016.[4]

Patients with diabetes develop common macro- and microvascular complications that result in an increased cardiovascular disease (CVD) risk.[5] As both diabetes and CVD are positioned in the top ten leading causes of death, stratification of patients with diabetes according to their CVD risk and proper management has become an essential need for health care providers. However, identifying which factors and interventions impact the course of the disease is not straightforward, because their impact can differ among cohorts depending on the socioeconomic context, on the health care provider practices and also because of the differences in the etiology of Type 1 and Type 2 diabetes.[6,7] Focusing on this need, several cardiovascular prediction models have been proposed over the years, some of them specifically designed for patients with diabetes.[8] Choosing the CVD risk model to be applied in a particular health system is not trivial, since external validations of the models are scarce and implementation procedures are rarely straightforward.

Taking advantage of the quality of the administrative and clinical datasets in Navarra, and our previous experiences of using some of these datasets[9], in 2016 we initiated the creation of the population-based CARDIANA (CARdiovscular risk in patients with DIAbetes in Navarra) cohort. To do

For peer review only - http://bmjopen.bmj.com/site/about/guidelines.xhtml

so, a longitudinal extraction from multiple health and administrative databases of all patients in Navarra with Type 1 (T1D) and Type 2 (T2D) diabetes was conducted under the Real Word Data (RWD) framework. The baseline and first 5-year follow-up data collection ended in 2017. The aims of setting up the CARDIANA cohort were: i) to establish a population-level dynamic cohort extraction and data integration mechanism that was nonexistent to date and could be used for research; ii) to assess which patient-level factors were determinant in the course of the disease in T1D and T2D patients of all ages, with a particular focus on socioeconomic factors; iii) to externally validate cardiovascular risk prediction models; and iv) to quantify the impact of health care provider and health care system actions on the CVD risk of this population.

Cohort description

This is a population-based cohort from Navarra, an autonomous community located in northern region of Spain with approximately 650000 inhabitants. The CARDIANA cohort includes all users of the Public Health Service of Navarra who, as of January 1st, 2012, had active codes of type 1 or type 2 diabetes (T89 and T90 of the International Classification of Primary Care, version 2, ICPC-2, respectively) in the Primary Care Electronic Medical Record System of Navarra (ATENEA) records. Patients with descriptions of diabetes different from T1D or T2D were excluded, as well as when severe inconsistencies in the dates of diagnosis, birth, or death were found. Patients were also excluded if no registry of contact with the public health system was found either before the inclusion date and/or in the follow-up period. No other exclusion criteria were applied, and patients of all ages and conditions were considered, including T2D patients with onset during childhood and T1D patients with late onset during adulthood. Causes of early termination of the patient data extraction were death or change of community/country.

For peer review only - http://bmjopen.bmj.com/site/about/guidelines.xhtml

Figure 1 shows the flowchart of the creation of the cohorts. The classification of patients into T1D or T2D took into account that the validity of the T2D diagnosis had been assessed in a previous study[10] but not that of the T1D diagnosis. Hence, the ICPC-2 codes and the descriptive field that goes with the code were first used, and after that, the classification procedure was complemented with the regional registry of T1D diabetes, which was legally approved by formal order 37/2014, on April 16th[11], that includes all T1D patients with an onset date after 1989. Combining the information from the health electronic records and administrative population datasets, two cohorts of prevalent patients with diabetes were created: the T1D CARDIANA cohort, with 1067 patients, and the T2D CARDIANA cohort, with 33842 patients. During the follow-up of the first wave, 33 (3.1%) T1D patients and 455 (1.3%) T2D patients were lost to follow-up due to reasons other than mortality.

The actual follow-up period of the cohort is five years, from January, 1st 2012, to December 31st, 2016. The next data extraction process that will update longitudinal data and principal cardiovascular events will be conducted in the autumn of 2022, covering the period from January 1st, 2017, to December 31st, 2021, and further extractions are planned in 5 year waves.

Variables, databases, and integration process

Sociodemographic and clinical variables of the defined CARDIANA T1D and CARDIANA T2D cohorts came from eight clinical and administrative databases: ATENEA, LAKORA-TIS, LAMIA, HCI, HIS-LEIRE (including the Minimum Basic Data Set at hospital discharge - MBDS), the population registry, the mortality registry and the Type 1 diabetes registry. For future updates of the cohort, the Results Analysis Database of Navarra (BARDENA) will be used. A brief description of the variables and the original dataset they came from is given in Supplementary Table S1, and the original datasets are extensively described in Moulis et al., 2018.[12] In all, the information collected consists of all relevant structured data available from these sources generated during each contact of the patient with the

For peer review only - http://bmjopen.bmj.com/site/about/guidelines.xhtml

health system. One set of variables was collected once and was considered fixed during the follow-up. These include the date of entry and/or exit from the health system, demographic and socioeconomic data such as the study level, lifestyle information such as tobacco use or physical activity level, the basic health zone the patients belongs to, coinsurance status[13], baseline comorbidities and past history of cardiovascular history using the ICPC-2 codification system, among others. Some new variables were created from these previous variables, such as the Charlson weighted score[14] or the GMA[15,16] comorbidity score. The other set of variables was collected longitudinally using a time-dependent structure and included all analytic results that occurred during follow-up as well as pharmacologic treatments, health service use and fatal and nonfatal cardiovascular events. For these time-dependent variables, the date on which they occurred was also included. Cardiovascular events were considered to occur during the follow-up when CVD diagnostic codes were recorded in the mortality or the MBDS dataset, adapted from the codes given in Read et al. (see the codes in Supplementary Table S1)[17].

The integration procedure was conducted by the Statistic Institute of Navarra (NASTAT) and the Directorate-General for Informatics, Telecommunications, and Innovation of the Health Department of Navarra, who supervised the data extraction and guaranteed fulfillment of the law in terms of personal data protection. Afterward, the anonymized databases were provided to the research team.

Findings to date

The T1D and T2D CARDIANA cohorts consisted of 1067 and 33842 patients, respectively. Their sociodemographic characteristics are given in Table 1. No adjustment has been included due to the descriptive nature of the objective, but information on both cohorts is presented in parallel. Patients in both cohorts were primarily men (57.4% and 55.7% in T1D and T2D cohorts respectively), and

For peer review only - http://bmjopen.bmj.com/site/about/guidelines.xhtml

only 5% were immigrants (compared to 12% in other Spanish regions)[18,19]. Compared to patients in the T2D cohort, patients in the T1D cohort were much younger (mean age 36.9 years in T1D vs. 69.4 years in T2D), had a higher probability of being working population (84.5% vs. 26.6%), had a higher income level (38.3% vs. 27.5% had over 18,000 € per year) and also higher educational level (17.8% vs. 4.7% had university studies).

Table 1: Demographic and socioeconomic characteristics of the T1D and T2DCARDIANA cohorts at baseline

	**T1D CARDIANA Cohort**			**T2D CARDIANA Cohort**		
	Male	Female	**Total**	Male	Female	**Total**
**n**	612	455	1067	18840	15002	33842
**Age**, Mean (SD)	36.6 (15.9)	37.2 (17.8)	36.9 (16.7)	67.1 (12.3)	72.3 (13.0)	69.4 (12.8)
**Working status, n(%)**						
Unemployed	36 (7.8)	21 (6.7)	57 (7.3)	836 (5.0)	609 (5.8)	1445 (5.3)
Working	392 (84.5)	264 (84.6)	656 (84.5)	5506 (33.1)	1713 (16.3)	7219 (26.6)
Pensioner	36 (7.8)	27 (8.7)	63 (8.1)	10308 (61.9)	8157 (77.8)	18465 (68.1)
(Missing)	*148*	*143*	*291*	*2190*	*4523*	*6713*
**Continent of origin, n(%)**						
Spain	559 (94.6)	415 (94.7)	974 (94.7)	17645 (95.9)	13853 (94.7)	31498 (95.3)
Europe	15 (2.5)	5 (1.1)	20 (1.9)	293 (1.6)	240 (1.6)	533 (1.6)
Africa	9 (1.5)	6 (1.4)	15 (1.5)	171 (0.9)	115 (0.8)	286 (0.9)
America	8 (1.4)	11 (2.5)	19 (1.8)	269 (1.5)	399 (2.7)	668 (2.0)
Asia	0 (0.0)	1 (0.2)	1 (0.1)	23 (0.1)	29 (0.2)	52 (0.2)
Australia	0 (0.0)	0 (0.0)	0 (0.0)	1 (0.0)	0 (0.0)	1 (0.0)
(Missing)	*21*	*17*	*38*	*438*	*366*	*804*
**Copayment category, n(%)**						
<18000	338 (56.4)	301 (68.9)	639 (61.7)	11445 (62.9)	12223 (84.6)	23668 (72.5)
≥18000	261 (43.6)	136 (31.1)	397 (38.3)	6747 (37.1)	2229 (15.4)	8976 (27.5)
(Missing)	13	18	31	648	550	1198
**Study level, n(%)**						
No formal education	132 (22.8)	79 (18.5)	211 (21.0)	5245 (28.6)	6033 (41.4)	11278 (34.3)
Primary School	226 (39.1)	157 (36.9)	383 (38.1)	9744 (53.1)	7410 (50.8)	17154 (52.1)
High School	136 (23.5)	95 (22.3)	231 (23.0)	2227 (12.1)	711 (4.9)	2938 (8.9)
University level	84 (14.5)	95 (22.3)	179 (17.8)	1121 (6.1)	424 (2.9)	1545 (4.7)
(Missing)	*34*	*29*	*63*	*503*	*424*	*927*
**Mean income, Mean (SD)** *(area level)*	12011.6 (1803.8)	12099.3 (1742.3)	12048.9 (1777.6)	11748.2 (1845.8)	11531.6 (1739.5)	11652.3 (1802.7)
**Income Quintile, n(%)** *(area level)*						
[7300,10565)	128 (21.7)	78 (17.8)	206 (20.0)	3539 (19.2)	3144 (21.5)	6683 (20.2)

For peer review only - http://bmjopen.bmj.com/site/about/guidelines.xhtml

	T1D CARDIANA Cohort			T2D CARDIANA Cohort		
	Male	Female	**Total**	Male	Female	**Total**
[10565,11416)	110 (18.6)	97 (22.1)	207 (20.1)	3515 (19.1)	3099 (21.2)	6614 (20.0)
[11416,12240)	122 (20.6)	84 (19.2)	206 (20.0)	3577 (19.4)	2980 (20.4)	6557 (19.8)
[12240,13394)	118 (20.0)	88 (20.1)	206 (20.0)	3820 (20.8)	2799 (19.1)	6619 (20.0)
[13394,17708]	113 (19.1)	91 (20.8)	204 (19.8)	3949 (21.5)	2615 (17.9)	6564 (19.9)
(Missing)	*21*	*17*	*38*	*440*	*365*	*805*

Percentage for each category are column percentages (% of patients in each category for each cohort), unless otherwise indicated (mean and standard deviations- SD are given in quantitative variables)

For peer review only

Health related patients' status at baseline, including lifestyle data, laboratory tests values, office measured parameters and comorbidities are given in Table 2. The mean time patients had been living with diabetes was three years higher in T1D patients than in T2D patients (11.0 vs. 8.1 years), but their comorbidity indices were lower, with a weighted Charlson score equal to 1.2 vs. 2.1 and a weighted GMA equal to 6.0 vs. 11.4, respectively. Similarly, T1D patients have much lower prevalence of cardiovascular disease history (4.9% vs. 23.8%), are more frequently active (71.5% vs 55.9%) and alcohol abstinent (69.0% vs 66.5%), but have higher probability of being smokers (32.2% vs 17.7%).

Table 2: Clinical and lifestyle characteristics of the T1D and T2D CARDIANA cohorts at baseline

		T1D CARDIANA Cohort			T2D CARDIANA Cohort		
		Male	Female	**Total**	Male	Female	**Total**
**n**		612	455	1067	18840	15002	33842
Clinical parameters, mean (SD)							
Duration of diabetes (years)		10.8 (9.2)	11.1 (9.1)	11.0 (9.1)	7.8 (5.8)	8.5 (6.3)	8.1 (6.0)
Body Mass Index (Kg/m2)		26.2 (4.5)	25.2 (5.0)	25.7 (4.7)	30.2 (5.3)	31.0 (6.4)	30.6 (5.8)
Systolic Blood Pressure (mm Hg)		124.0(19.0)	121.4(19.9)	122.9(19.4)	135.5 (16.9)	135.7 (17.9)	135.6 (17.3)
Diastolic Blood Pressure (mm Hg)		72.3 (12.3)	71.0 (9.8)	71.7 (11.2)	76.6 (10.5)	75.8 (10.5)	76.2 (10.5)
Laboratory tests, mean (SD)							
HbA1c (%)		8.2 (1.6)	8.2 (1.4)	8.2 (1.5)	7.0 (1.3)	7.1 (1.3)	7.1 (1.3)
Fasting glucose (mg/dL)		179.9(97.6)	172(80.5)	176.3(89.3)	141.6 (44.6)	139.0 (45.4)	140.4 (45.0)
Total Cholesterol (mg/dL)		188(42.1)	188(31.2)	188(36.9)	183.2 (39.2)	194.0 (37.9)	188.0 (39.0)
High Density Lipoprotein (mg/dL)		57.5 (17.0)	68.4 (17.8)	62.0 (18.2)	46.3 (13.3)	52.1 (14.4)	48.9 (14.1)
Low Density Lipoprotein (mg/dL)		107.2(29.8)	103.6(28.7)	105.7(29.4)	108.9 (31.9)	114.4 (32.4)	111.4 (32.2)
Triglicerides (mg/dL)		90.7 (58.5)	75.1 (37.7)	84.2 (51.4)	141.5 (77.3)	144.1 (68.9)	142.7 (73.7)
Creatinine (mg/dL)		0.9 (0.3)	0.7 (0.2)	0.8 (0.3)	1.1 (0.6)	0.9 (0.5)	1.0 (0.6)
Albumin to creatinine ratio		12.9 (30.9)	14.0 (31.5)	13.4 (31.2)	40.2 (165.6)	30.4 (134.0)	35.9 (152.4)
Lifestyle data, n(%)							
Smoking Status	Non Smoker	149 (49.2)	150 (60.2)	299 (54.2)	6009 (37.9)	11025(85.1)	17034(59.1)

For peer review only - http://bmjopen.bmj.com/site/about/guidelines.xhtml

		T1D CARDIANA Cohort			T2D CARDIANA Cohort		
		Male	Female	**Total**	Male	Female	**Total**
	Ex-smoker	51 (16.8)	24 (9.6)	75 (13.6)	5810 (36.6)	887 (6.8)	6697 (23.2)
	Smoker	103 (34.0)	75 (30.1)	178 (32.2)	4052 (25.5)	1041 (8.0)	5093 (17.7)
	(Missing)	*309*	*206*	*515*	*2969*	*2049*	*5018*
Alcohol	Abstinent	150 (59.5)	159 (81.1)	309 (69.0)	7082 (47.5)	10805(90.1)	17887(66.5)
	Moderate drinker	95 (37.7)	37 (18.9)	132 (29.5)	7044 (47.3)	1137 (9.5)	8181 (30.4)
	Heavy drinker	7 (2.8)	0 (0.0)	7 (1.6)	777 (5.2)	44 (0.4)	821 (3.1)
	(Missing)	*360*	*259*	*619*	*3937*	*3016*	*6953*
	Physical activity						
	Inactive	7 (4.0)	11 (7.6)	18 (5.6)	1431 (9.8)	1931 (16.1)	3362 (12.6)
	Partially active	38 (21.7)	35 (24.3)	73 (22.9)	3966 (27.2)	4405 (36.8)	8371 (31.5)
	Active	130 (74.3)	98 (68.1)	228 (71.5)	9207 (63.0)	5647(47.1)	14854(55.9)
	(Missing)	*437*	*311*	*748*	*4236*	*3019*	*7255*
Comorbidities							
Charlson score, mean (SD)		1.2 (0.8)	1.2 (0.8)	1.2 (0.8)	2.2 (1.7)	2.1 (1.6)	2.1 (1.7)
GMA[a] score, mean (SD)		5.5 (3.8)	6.7 (4.7)	6.0 (4.2)	10.7 (5.8)	12.2 (5.9)	11.4 (5.9)
Previous CVD		31 (5.1)	21 (4.6)	52 (4.9)	4804 (25.5)	3236 (21.6)	8040 (23.8)
Diabetes related comorbidities, n(%)							
Retinopathy (%)	Yes	25 (35.2)	11 (18.6)	36 (27.7)	670 (15.3)	616 (18.9)	1286 (16.8)
	(Missing)	*541*	*396*	*937*	*14451*	*11738*	*26189*
Amputation	Yes	1 (1.2)	0 (0.0)	1 (0.7)	160 (2.0)	54 (0.8)	214 (1.5)
	(Missing)	*532*	*390*	*922*	*10865*	*8252*	*19117*
Diabetic foot risk	None	4 (5.9)	4 (6.2)	8 (6.0)	230 (3.2)	212 (3.5)	442 (3.3)
	Superficial ulcer	53 (77.9)	56 (86.2)	109 (82.0)	6048 (83.6)	4809 (79.7)	10857(81.8)
	Deep tissue ulcers w/o abcess	8 (11.8)	3 (4.6)	11 (8.3)	652 (9.0)	704 (11.7)	1356 (10.2)
	Deep tissue ulcers w abcess	2 (2.9)	1 (1.5)	3 (2.3)	182 (2.5)	244 (4.0)	426 (3.2)
	Localized gangrene	1 (1.5)	1 (1.5)	2 (1.5)	113 (1.6)	62 (1.0)	175 (1.3)
	Extensive gangrene	0 (0.0)	0 (0.0)	0 (0.0)	10 (0.1)	4 (0.1)	14 (0.1)
	(Missing)	*544*	*390*	*934*	*11605*	*8967*	*20572*
Cardiovascular risk scores, mean (SD)							
SCORE[b], mean (SD)		1.9 (2.6)	0.8 (1.2)	1.4 (2.1)	4.9 (6.6)	2.0 (1.7)	3.6 (5.3)

Percentage for each category are column percentages (% of patients in each category for each cohort) unless otherwise indicated (mean and standard deviations- SD are given in quantitative variables)
[a]GMA: Adjusted Morbidity groups ;[b]SCORE: Systematic COronary Risk Evaluation

Regarding clinical and laboratory test parameters, T1D patients showed much better control of their body mass index (25.7 vs. 30.6 kg/m^2) but worse glycemic control of their HbA1c levels (8.2% vs. 7.1%). They also had better control of the other metabolic parameters considered, namely, high-density lipoprotein (62.0 vs. 48.9 mg/dL), low-density lipoprotein (105.7 vs. 111.4 mg/dL), triglycerides (84.2 vs. 142.7 mg/dL) and albumin to creatinine ratio levels (13.4 vs. 35.9).

The use of primary care services during the last year before baseline (Table 3) was lower in T1D than in T2D patients for the total number of visits (17.1 vs. 21.1 visits/year) and for all types of visits,

For peer review only - http://bmjopen.bmj.com/site/about/guidelines.xhtml

except for emergency visits (0.6 vs. 0.5 visits/year) and remote visits to nursing (4.1 vs. 1.1 visits/year). Active prescriptions of antihypertensive medication were 41 points less frequent in patients with T1D (15.2% vs. 56.2%), and their lipid-lowering treatment use was less than half (18.8% vs. 49.3%). Similar differences were observed in antithrombotic treatment prescription (15.6% vs 38.2%), but baseline glucose-lowering treatment prescription was higher in patients with T1D (83.6% vs. 67.2%).

Table 3: Use of Primary Care Services during the year previous to baseline and active prescriptions at baseline

	T1D CARDIANA Cohort			T2D CARDIANA Cohort		
	Male	Female	**Total**	Male	Female	**Total**
**n**	612	455	1067	18840	15002	33842
**Total visits,** mean (SD)	15.9 (11.4)	19.0 (12.6)	17.1 (12.2)	19.4 (19.1)	23.2 (20.8)	21.1 (19.9)
**Visits at Office, by professional,** mean(SD)						
Nursing	5.2 (6.7)	5.6 (6.9)	5.4 (6.8)	7.1 (10.3)	7.6 (9.7)	7.3 (10.1)
Physician	3.8 (4.3)	5.2 (5.5)	4.4 (4.9)	6.4 (5.8)	7.3 (6.5)	6.8 (6.1)
Social Worker	0.8 (1.4)	1.3 (3.4)	1.0 (2.5)	1.7 (2.4)	1.9 (3.6)	1.7 (3.0)
Emergency	0.1 (0.3)	0.1 (0.5)	0.1 (0.4)	0.1 (0.6)	0.2 (0.9)	0.1 (0.8)
Other	0.6 (1.7)	0.7 (1.5)	0.6 (1.6)	0.5 (2.7)	0.6 (1.8)	0.5 (2.3)
**Visits at home, by professional,** mean(SD)						
Nursing	0.1 (0.8)	0.0 (0.4)	0.1 (0.7)	0.7 (5.7)	1.4 (7.8)	1.0 (6.7)
Physician	0.1 (0.3)	0.0 (0.2)	0.0 (0.3)	0.3 (1.7)	0.6 (2.2)	0.4 (1.9)
Social Worker	0.0 (0.1)	0.0 (0.2)	0.0 (0.2)	0.1 (1.2)	0.2 (1.6)	0.1 (1.4)
Emergency	0.0 (0.0)	0.0 (0.1)	0.0 (0.0)	0.0 (0.1)	0.0 (0.2)	0.0 (0.2)
Other	0.0 (0.3)	0.0 (0.1)	0.0 (0.3)	0.1 (0.6)	0.1 (0.8)	0.1 (0.7)
**Remote visits, by professional,** mean(SD)						
Nursing	3.9 (4.9)	4.3 (5.6)	4.1 (5.2)	0.9 (2.3)	1.3 (2.8)	1.1 (2.6)
Physician	1.4 (2.3)	1.8 (2.9)	1.5 (2.6)	1.5 (3.1)	2.0 (3.9)	1.7 (3.5)
Social Worker	0.0 (0.1)	0.0 (0.1)	0.0 (0.1)	0.0 (0.2)	0.0 (0.2)	0.0 (0.2)
Emergency	0.0 (0.2)	0.0 (0.2)	0.0 (0.2)	0.0 (0.4)	0.1 (0.6)	0.1 (0.5)
**Drug treatments use,** n(%)						
Antihypertensive	95 (15.5)	67 (14.7)	162 (15.2)	10303(54.7)	8769(58.5)	19072(56.4)
Glucose-lowering	519 (84.8)	373 (82.0)	892 (83.6)	12630(67.0)	10099(67.3)	22729(67.2)
Lipid-lowering	122 (19.9)	79 (17.4)	201 (18.8)	9423(50.0)	7249(48.3)	16672(49.3)
Antithrombotic	67 (10.9)	47 (10.3)	114 (10.7)	7722(41.0)	5262(35.1)	12984(38.4)

Percentage for each category are row percentages (% of patients with each treatment in each cohort), unless otherwise indicated (mean and standard deviations- SD are given in quantitative variables)

Only 17 patients (1.6%; 95% CI [1.0, 2.5]) in the T1D cohort developed a CVD event, five of which were fatal (0.46% of the total cohort), whereas for the T2D cohort, 2882 (8.5%; 95% CI [8.2, 8.8]) had an event and 1307 of them were fatal (3.9% of the total cohort). During the follow up, 20

For peer review only - http://bmjopen.bmj.com/site/about/guidelines.xhtml

patients with T1D died while 6378 patients with T2D died in the same period. For the T2D cohort, the occurrence of CVD events along the follow-up has been associated with physical activity, with estimated ORs after matching and adjusting for confounders equal to 0.84 (95% CI: 0.66-1.07) for the partially active group and 0.71 (95% CI: 0.56-0.91) for the active group, compared with patients in the non-active group. [20]

Strengths and limitations

The main strength of this study is that the CARDIANA cohorts integrate exhaustive clinical, socioeconomic and behavioral information from all available administrative and clinical data sources, providing a complete framework to assess the course of the disease in patients with diabetes and the factors that affect it. Especially in relation to socioeconomic variables, these cohorts have individual information on country of origin, working status, educational level and income, which are not frequently available, with most studies using area-level proxies. Another strength is that data have been subjected to quality control procedures before and after database integration.

The main limitation of this study is the possible presence of bias resulting from the use of existing electronic clinical records, which may affect different methodological aspects. First, T2D patients without a diabetes code in the ATENEA records were not included. Although the validity of the code has been satisfactory asssessed,[10] undiagnosed patients have not been included, which in Spain it has been estimated that could account for 4-6% of the overall prevalence of 11-14%.[21,22] Second, data completeness can be low for some variables dependent on physicians' idiosyncratic reporting procedures, such as tobacco use or physical activity. Although an effort has been made to complement variables with others that had a text format, information bias may be present, so imputation methods and sensitivity analyses will be required. Third, electronic prescriptions were only fully implemented in 2014, and at baseline it is estimated that 8-10% of the total prescriptions

For peer review only - http://bmjopen.bmj.com/site/about/guidelines.xhtml

have not been accounted for. Fourth, patients without any contact with the regional public health system because of using exclusively private health institutions were not included. Nevertheless, it is estimated that these patients account for only 3.2% of the total population[23].

Collaboration

Requests for collaborative studies are welcome, upon request with a description of the planned projects from berta.ibanez.beroiz@navarra.es. They will only be considered after the approval of the research ethics committee from the solicitor institution and from the Navarra health system – Osasunbidea and the NASTAT institutions – responsible for the clinical information and the population information.

Patient and Public Involvement

The rationale behind the cohort was to gather information to inform and assess initiatives aimed at improving the health care provided for patients with Type 1 and Type 2 Diabetes. To do so, the CARDIANA cohorts were designed by a multidisciplinary team involving methodologist, primary care specialist, endocrinologist, health care policy makers and clinical and social science researchers, many of them involved in the design of strategies for the management of patients with diabetes. The selection and definition of the variables considered and the prioritization of research questions were conducted by agreement with the research team. Additionally, the creation of these cohorts was used as 'case study' in the development of BARDENA, the Results Analysis Database of Navarra that is being constructed under the adoption of the Observational Medical Outcomes Partnership (OMOP) Common Data Model, which aims at harmonizing electronic medical records to facilitate participation on international distributed research.

For peer review only - http://bmjopen.bmj.com/site/about/guidelines.xhtml

Ethics approval

The study protocol was favorably evaluated by the Ethics Committee of Clinical Research of Navarra (Project 2015/111). This study has a retrospective nature, and data were irreversibly anonymized prior to transfer to the research team. The study was conducted according to the amended Declaration of Helsinki, Organic Law 3/2018, the General Data Protection Regulation (EU) 2016/679 and International Guidelines for Ethical Review of Epidemiological Studies.

Author contributions

BI and KC designed the study, researched the data and reviewed the manuscript. IT analyzed the data and wrote the manuscript. JLi, AG and ME researched the data and reviewed the manuscript. LF and MJG participated in the design of the study, in the acquisition of the T1D registry data and reviewed the manuscript. OL, JG, and AO participated in the acquisition and validation of the ATENEA, LAKORA, LAMIA, HIS-Leire and HCI datasets and reviewed the manuscript. LA participated in the acquisition and validation of the population registry and reviewed the manuscript. CM participated in the acquisition and validation of the mortality registry database and reviewed the manuscript. JLa participated in the design of the study, contributed to the interpretation of the results and reviewed the manuscript. All authors have approved this version of the manuscript.

Supplementary data

Supplementary data are available at BMJ open online.

Funding

This work has received funding from the Instituto de Salud Carlos III through grant PI15/02196 and via the CONCEPT project (grants PI19/00154, PI19/00381). It has also received funding from

For peer review only

For peer review only - http://bmjopen.bmj.com/site/about/guidelines.xhtml

REDISSEC RD16/0001/0014 and RICAPPS RD21/0016/0016, two Spanish Networks supported by Carlos III Health Institute and the European Regional Development Funding (FEDER). All funds are public and had no role in the design of the study, analysis or writing of the paper.

Acknowledgements

The authors thank all professionals who participated in the acquisition of the data, especially Javier Baquedano, Adriana Rivero, Mª Ángeles Nuin and Alberto Jiménez-Idiazábal. The authors belong to the group RD16/0001/0014 of REDISSEC and to the group RD21/0016/0016 of RICAPPS.

Conflict of interest

None declared.

Figures

Figure 1: Type 1 and type 2 diabetes prevalent cohort creation flowchart

References

1. International Diabetes Federation. IDF Diabetes Atlas, 9th edn. [Internet]. Brussels,Belgium; 2019. p. 476. Available from: https://www.diabetesatlas.org

2. Alberti K, Zimmet P. Definition, diagnosis and classification of diabetes mellitus and its complications. Part 1: diagnosis and classification of diabetes mellitus report of a WHO consultation. *Diabet Med*. 1998;**15**(7):539–53.

3. The Lancet. Turning evidence into action on diabetes. *Lancet* [Internet]. Elsevier Ltd; 2020;**396**(10262):1535. Available from: http://dx.doi.org/10.1016/S0140-6736(20)32412-0

4. Dain K. Data-driven policies needed to turn the tide on diabetes. *Lancet* [Internet]. Elsevier

For peer review only - http://bmjopen.bmj.com/site/about/guidelines.xhtml

Ltd; 2020;**396**(10267):1948–1949. Available from: http://dx.doi.org/10.1016/S0140-6736(20)32378-3

5. Peters SAE, Huxley RR, Sattar N, Woodward M. Sex Differences in the Excess Risk of Cardiovascular Diseases Associated with Type 2 Diabetes: Potential Explanations and Clinical Implications. *Curr Cardiovasc Risk Rep*. 2015;**9**(7):1–7.

6. Livingstone SJ, Looker HC, Hothersall EJ, et al. Risk of Cardiovascular Disease and Total Mortality in Adults with Type 1 Diabetes: Scottish Registry Linkage Study. *PLoS Med*. 2012;**9**(10).

7. Forbes JM, Cooper ME. Mechanisms of diabetic complications. *Physiol Rev*. 2013;**93**(1):137–188.

8. Galbete A, Tamayo I, Librero J, Enguita-Germán M, Cambra K, Ibáñez-Beroiz B. Cardiovascular risk in patients with type 2 diabetes: A systematic review of prediction models. *Diabetes Res Clin Pract*. 2022;**184**.

9. Ibáñez B, Galbete A, Goñi MJ, et al. Socioeconomic inequalities in cardiometabolic control in patients with type 2 diabetes. *BMC Public Health*. BMC Public Health; 2018;**18**(1):1–9.

10. Moreno-Iribas C, Sayon-Orea C, Delfrade J, et al. Validity of type 2 diabetes diagnosis in a population-based electronic health record database. *BMC Med Inform Decis Mak*. BMC Medical Informatics and Decision Making; 2017;**17**(1):1–6.

11. Orden Foral 37/2014. Navarra, Spain: https://bon.navarra.es/es/anuncio/-/texto/2014/88/10; 2014.

12. Moulis G, Ibañez B, Palmaro A, et al. Cross-national health care database utilization

For peer review only - http://bmjopen.bmj.com/site/about/guidelines.xhtml

between spain and france: results from the epichronic study assessing the prevalence of type 2 diabetes mellitus. *Clin Epidemiol*. 2018;**10**:863–874.

13. Pozo-Rubio R del, Pardo-García I, Escribano-Sotos F. El copago de dependencia en España a partir de la reforma estructural de 2012. *Gac Sanit*. 2017;**31**(1):23–29.

14. Charlson ME, Pompei P, Ales KL, MacKenzie CR. A new method of classifying prognostic comorbidity in longitudinal studies: development and validation. *J Chronic Dis*. England; 1987;**40**(5):373–383.

15. González González AIG, Miquel Gómez AM, Rodríguez Morales D, et al. Concordancia y utilidad de un sistema de estratificación para la toma de decisiones clínicas. *Atención Primaria*. 2017;**49**(4):240–247.

16. Monterde D, Vela E, Clèries M. Adjusted morbidity groups: A new multiple morbidity measurement of use in Primary Care. *Aten Primaria* [Internet]. Elsevier España, S.L.U.; 2016;**48**(10):674–682. Available from: http://dx.doi.org/10.1016/j.aprim.2016.06.003

17. Read SH, Diepen M Van, Colhoun HM, et al. Performance of cardiovascular disease risk scores in people diagnosed with type 2 diabetes: External validation using data from the National Scottish diabetes register. *Diabetes Care*. 2018;**41**(9):2010–2018.

18. Rodríguez-Sanz M, Gotsens M, Marí Dell'Olmo M, Borrell C. Trends in mortality inequalities in an urban area: The influence of immigration. *Int J Equity Health*. International Journal for Equity in Health; 2019;**18**(1):1–9.

19. Oliva J, Pérez G. Inmigración y salud. *Gac Sanit*. 2009;**23**(SUPPL. 1):1–3.

For peer review only - http://bmjopen.bmj.com/site/about/guidelines.xhtml

20. Enguita-Germán M, Tamayo I, Galbete A, Librero J, Cambra K, Ibáñez-Beroiz B. Effect of physical activity on cardiovascular event risk in a population-based cohort of patients with type 2 diabetes. *Int J Environ Res Public Health*. 2021;**18**(23).

21. Soriguer F, Goday A, Bosch-Comas A, et al. Prevalence of diabetes mellitus and impaired glucose regulation in Spain: The Di@bet.es Study. *Diabetologia*. 2012;**55**(1):88–93.

22. Aguayo A, Urrutia I, González-Frutos T, et al. Prevalence of diabetes mellitus and impaired glucose metabolism in the adult population of the Basque Country, Spain. *Diabet Med*. 2017;**34**(5):662–666.

23. Ministerio de Sanidad y Consumo. Encuesta Nacional de Salud 2011-2012 [Internet]. Available from: https://www.mscbs.gob.es/estadEstudios/estadisticas/encuestaNacional/encuestaNac2011/UtilizacionServiciosSanitarios_DistribucionPorcentual.pdf

For peer review only

For peer review only - http://bmjopen.bmj.com/site/about/guidelines.xhtml

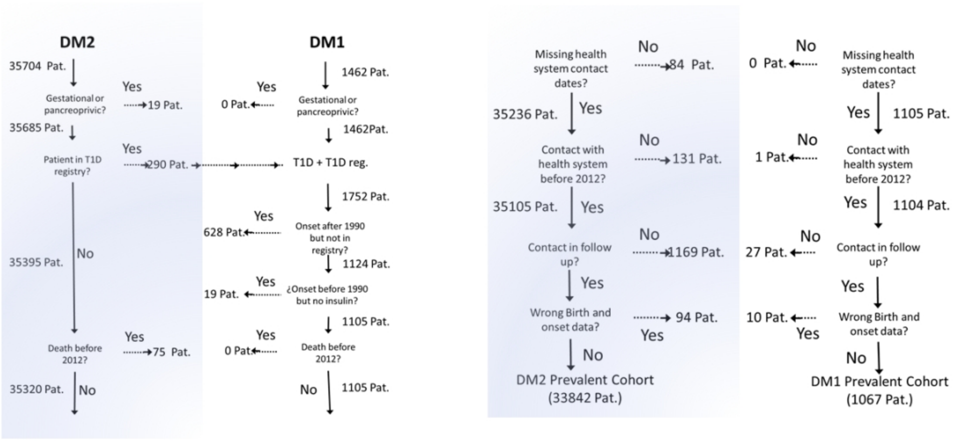

Type 1 and type 2 diabetes prevalent cohort creation flowchart

338x190mm (96 x 96 DPI)

Supplementary Table S1: Data information available in the T1D and T2D cohorts, by type of information and data source

Data Source[Ω]	Variables
**Socioeconomic data**	
Population Registry	Date of birth, country of origin, study level, marital status, working status and mean income of the assigned basic health zone
LAKORA-TIS	Pharmaceutical co-payment category
ATENEA-TIS	Date of birth, date of inclusion/withdrawal in the Navarra health system, general practitioner identification
**Previous diagnosis and past history of diseases**	
ATENEA	Weighted GMA, Past-history of myocardial infarction, congestive heart failure, cerebrovascular disease, peripheral vascular disease, retinopathy, neuropathy, nephropathy, foot lesions, dementia, chronic pulmonary disease, rheumatic disease, peptic ulcer disease , hemiplegia or paraplegia , neoplasm, liver disease , AIDS/HIV.Past history of CVD events coded in one of the following ICPC-2 codes: K74, K75, K76, K77, K89, K90, K91
**Personal cardiovascular risk factors, laboratory test and lifestyle behavior**	
ATENEA	Sex, age, physical activity, alcohol consumption, smoking status, self-management of pharmacological treatment, time since diagnosis, office, and laboratory test parameters (and dates): weight, height, systolic blood pressure (SBP), diastolic blood pressure (DBP), Total Cholesterol, LDL, HDL, triglycerides, creatinine, haemoglobin, glycated haemoglobin, fasting glucose, glomerular filtration rate (GFR), urine albumin to creatinine ratio.
The HCI database	Data collected by specialist care units: office and laboratory test parameters and procedures (date and value)
**Use of health services**	
HIS-LEIRE	Hospital or emergency room admissions, diagnostics, procedures and dates of admission and discharge
ATENEA	Number of yearly telephonic/physical consultation, office/home-based consultation and type of service provided (nurse/ medical/social worker/Emergency room)
**Drug treatment use**	
LAMIA	After 2013: Treatment ATC code, date of prescription and date of dispensation
ATENEA	Before 2013: Treatment ATC code and date of prescription
**Diabetes classification**	
T1D registry	Type 1 diabetes confirmation and onset date
**Outcomes**	
Mortality registry	Cause of death and date
HIS-LEIRE	Minimum Basic Data Set (MBDS): ICD-9CM until December 31st, 2015 and ICD-10-ES from January 1st, 2016. Hospitalization or death related to a cardiovascular event coded in one of the following: ICD-9 codes: 410,430,431,432,433,434,435;ICD-10 codes: I20-I25, I46,I60-I63,I65,G45

[Ω] For future updates of the cohort, the Results Analysis Database of Navarre (BARDENA) will be used

BMJ Open

Cohort Profile: CArdiovascular Risk in patients with DIAbetes in Navarra (CARDIANA cohort)

Journal:	*BMJ Open*
Manuscript ID	bmjopen-2022-066052.R1
Article Type:	Cohort profile
Date Submitted by the Author:	01-Dec-2022

Complete List of Authors:	Tamayo, Ibai; Navarrabiomed, IdiSNA, Unidad de Metodología; Red de Investigación en Cronicidad, Atención Primaria y Promoción de la Salud<br>Librero-Lopez, Julian ; Navarrabiomed, IdiSNA; Red de Investigacion en Servicios de Salud en Enfermedades Cronicas, Red de Investigación en Cronicidad, Atención Primaria y Servicios de Salud<br>Galbete, Arkaitz; Universidad Pública de Navarra, Estadística; Navarrabiomed, IdISNA<br>Cambra, Koldo; Departamento de Sanidad. Gobierno Vasco.<br>Enguita, Mónica; Navarrabiomed, IdiSNA; Red de Investigación en Servicios de Salud en Enfermedades Crónicas, Red de Investigación en Cronicidad, Atención Primaria y Promoción de la Salud<br>Forga, Luis; Servicio Navarro de Salud - Osasunbidea, Servicio de Endocrinología y Nutrición - HUN; Red de Investigación en Servicios de Salud en Enfermedades Crónicas, Red de Investigación en Cronicidad, Atención Primaria y Promoción de la Salud<br>Goñi, José; Servicio Navarro de Salud - Osasunbidea, Servicio de Endocrinología y Nutrición - HUN; Red de Investigación en Servicios de Salud en Enfermedades Crónicas, Red de Investigación en Cronicidad, Atención Primaria y Promoción de la Salud<br>Lecea, Oscar; Servicio Navarro de Salud - Osasunbidea, Atención Primaria; Red de Investigación en Servicios de Salud en Enfermedades Crónicas, Red de Investigación en Cronicidad, Atención Primaria y Promoción de la Salud<br>Gorricho, Javier; Servicio Navarro de Salud - Osasunbidea, Servicio de Evaluación y Difusión de resultados en Salud; Red de Investigacion en Servicios de Salud en Enfermedades Cronicas, Red de Investigación en Cronicidad, Atención Primaria y Servicios Sanitarios<br>Olazarán, Álvaro; Departamento de Universidad, Innovación y Transformación, Servicio Tecnologías de la Salud<br>Arnedo, Laura; Instituto de Estadística de Navarra<br>Moreno-Iribas , Conchi; Instituto de Salud Pública y Laboral de Navarra; Red de Investigacion en Servicios de Salud en Enfermedades Cronicas, Red de Investigación en Cronicidad, Atención Primaria y Promoción de la Salud<br>Lafita, Javier; Servicio Navarro de Salud - Osasunbidea, Servicio de Efectividad y Seguridad Asistencial ; Red de Investigación en Servicios de Salud en Enfermedades Crónicas, Red de Investigación en Cronicidad, Atención Primaria y Promoción de la Salud<br>Ibañez, Berta; Navarrabiomed, IdiSNA; Red de Investigacion en Servicios de Salud en Enfermedades Cronicas, Red de Investigación en Cronicidad, Atención Primaria y Promoción de la Salud

**Primary Subject Heading**:	Epidemiology
Secondary Subject Heading:	Diabetes and endocrinology, Cardiovascular medicine
Keywords:	General diabetes < DIABETES & ENDOCRINOLOGY, Cardiac Epidemiology < CARDIOLOGY, Risk management < HEALTH SERVICES ADMINISTRATION & MANAGEMENT, Health informatics < BIOTECHNOLOGY & BIOINFORMATICS

SCHOLARONE™ Manuscripts

I, the Submitting Author has the right to grant and does grant on behalf of all authors of the Work (as defined in the below author licence), an exclusive licence and/or a non-exclusive licence for contributions from authors who are: i) UK Crown employees; ii) where BMJ has agreed a CC-BY licence shall apply, and/or iii) in accordance with the terms applicable for US Federal Government officers or employees acting as part of their official duties; on a worldwide, perpetual, irrevocable, royalty-free basis to BMJ Publishing Group Ltd ("BMJ") its licensees and where the relevant Journal is co-owned by BMJ to the co-owners of the Journal, to publish the Work in this journal and any other BMJ products and to exploit all rights, as set out in our licence.

The Submitting Author accepts and understands that any supply made under these terms is made by BMJ to the Submitting Author unless you are acting as an employee on behalf of your employer or a postgraduate student of an affiliated institution which is paying any applicable article publishing charge ("APC") for Open Access articles. Where the Submitting Author wishes to make the Work available on an Open Access basis (and intends to pay the relevant APC), the terms of reuse of such Open Access shall be governed by a Creative Commons licence – details of these licences and which Creative Commons licence will apply to this Work are set out in our licence referred to above.

Other than as permitted in any relevant BMJ Author's Self Archiving Policies, I confirm this Work has not been accepted for publication elsewhere, is not being considered for publication elsewhere and does not duplicate material already published. I confirm all authors consent to publication of this Work and authorise the granting of this licence.

For peer review only

TITLE PAGE

Cohort Profile: CArdiovascular Risk in patients with DIAbetes in Navarra (CARDIANA cohort)

Ibai Tamayo[a,b] , Julián Librero[a,b], Arkaitz Galbete[a,c], Koldo Cambra[b,d], Mónica Enguita[a,b], Luis Forga[b,e], Mª José Goñi[b,e], Oscar Lecea[b,f], Javier Gorricho[b,g], Álvaro Olazarán[h], Laura Arnedo[i], Conchi Moreno-Iribas[b,j], Javier Lafita[b,k] and Berta Ibáñez-Beroiz *[a,b]

a. Unidad de Metodología. Navarrabiomed-Hospital Universitario de Navarra-Universidad Pública de Navarra. IdiSNA. Pamplona. Spain
b. Red de Investigación en Servicios Sanitarios y Enfermedades Crónicas (REDISSEC) y Red de Investigación en Cronicidad, Atención Primaria y Promoción de la Salud (RICAPPS). Pamplona. Spain
c. Departamento de Estadística. Universidad Pública de Navarra (UPNA). Pamplona. Spain
d. Dirección de Salud Pública y Adicciones. Departamento de Sanidad. Gobierno Vasco. Vitoria. Spain
e. Servicio de Endocrinología y Nutrición, Hospital Universitario de Navarra, Servicio Navarro de Salud (SNS-O). Pamplona. Spain
f. Atención Primaria, Servicio Navarro de Salud (SNS-O). Pamplona. Spain
g. Servicio de Evaluación y Difusión de resultados en Salud, Servicio Navarro de Salud (SNS-O). Pamplona. Spain
h. Servicio Tecnologías de la Salud, Dpto de Universidad, Innovación y Transformación. Pamplona. Spain
i. Instituto de Estadística de Navarra (NASTAT). Pamplona. Spain
j. Instituto de Salud Pública y Laboral de Navarra (ISPLN). Pamplona. Spain
k. Servicio de Efectividad y Seguridad Asistencial (SNS-O). Pamplona. Spain

For peer review only

For peer review only - http://bmjopen.bmj.com/site/about/guidelines.xhtml

ibai.tamayo.rodriguez@navarra.es

julian.librero.lopez@navarra.es

arkaitz.galbete@unavarra.es

k-cambra@euskadi.eus

monica.enguita.german@navarra.es

lluis.forga.llenas@navarra.es

mj.goni.iriarte@navarra.es

oscar.lecea.juarez@navarra.es

javier.gorricho.mendivil@navarra.es

alvaro.olazaran.santesteban@navarra.es

laura.arnedo.ajona@navarra.es

mc.moreno.iribas@navarra.es

javier.lafita.tejedor@navarra.es

CORRESPONDING AUTHOR: Berta Ibáñez-Beroiz berta.ibanez.beroiz@navarra.es

Navarrabiomed-Complejo Hospitalario de Navarra-Universidad Pública de Navarra

C/ Irunlarrea s/n. Recinto CHN. 31008. Pamplona. Spain Tfno: 0034848428393;

Word count: 2759

For peer review only - http://bmjopen.bmj.com/site/about/guidelines.xhtml

Abstract

Purpose: The CARDIANA cohort was established to assess the effects of sociodemographic and clinical variables on the risk of cardiovascular events in patients with type 1 (T1D) or type 2(T2D) diabetes, with a special focus on socioeconomic factors, and to validate and develop cardiovascular risk models for these patients.

Participants: The CARDIANA cohort included all patients with type 1 (T1D) and type 2 (T2D) diabetes registered in the Public Health Service of Navarra with prevalent disease on January 1, 2012. It consisted of 1067 T1D patients (ages 2-88 years) and 33842 T2D patients (ages 20-105 years), whose data were retrospectively extracted from the Health and Administrative System Databases.

Findings to date: The follow-up period for Wave 1 was from January 1, 2012 to December 31, 2016. During these five years, 17 patients (1.6%; 95% CI [1.0, 2.5]) in the T1D cohort developed a CVD event, whereas for the T2D cohort, 2882 (8.5%; 95% CI [8.2, 8.8]) had an event. For the T2D cohort, physical activity was associated with a reduced risk of cardiovascular events, with adjusted estimated odds ratios equal to 0.84 (95% CI: 0.66-1.07) for the partially active group and 0.71 (95% CI: 0.56-0.91) for the active group, compared with patients in the non-active group.

Future plans: The CARDIANA cohort is currently being used to assess the effect of sociodemographic risk factors on CV risk at 5 years and to externally validate cardiovascular predictive models. A second Wave will be conducted at the end of 2022, to extend the follow-up other five years, from January 1, 2016 to December 31, 2021. Periodic data extractions are planned every five year.

For peer review only - http://bmjopen.bmj.com/site/about/guidelines.xhtml

Strengths and limitations of this study

- The CARDIANA cohorts integrate exhaustive clinical, socioeconomic and behavioral information from all available administrative and clinical data sources in patients with T1D and T2D diabetes.
- The data have been subject to quality control procedures before and after database integration.
- The presence of possible bias resulting from the use of existing electronic clinical records require to be accounted for.
- Some variables may be underreported, and electronic prescriptions were only fully implemented in 2014.

For peer review only - http://bmjopen.bmj.com/site/about/guidelines.xhtml

Introduction

Diabetes mellitus is a common metabolic disorder that affected one in ten adults worldwide in 2021. Approximately 11.5% of the total health care spending and 12.2% of global all-cause deaths in adults aged 20-79 years are attributable to diabetes.[1] Despite governments agreeing to halt the increase in diabetes and obesity by 2025,[2] projections for 2045 show a growth of 16% in the expected prevalence of diabetes, becoming one of the fastest growing global health emergency of the 21st century.[1]

Patients with diabetes develop common macro- and microvascular complications that result in an increased cardiovascular disease (CVD) risk.[3] Stratification of patients with diabetes according to their CVD risk and proper management has become an essential need for health care providers. However, identifying which factors and interventions impact the course of the disease is not straightforward, because their impact can differ among cohorts depending on the socioeconomic context, on the health care provider practices and also because of the differences in the etiology of Type 1 and Type 2 diabetes.[4,5] Focusing on this need, several cardiovascular prediction models have been proposed over the years, some of them specifically designed for patients with diabetes.[6] Choosing the CVD risk model to be applied in a particular health system is not trivial, since external validations of the models are scarce and implementation procedures are rarely straightforward.

Taking advantage of the quality of the administrative and clinical datasets in Navarra, already used for research in patients with Type 2 Diabetes,[7] we initiated the creation of the population-based CARDIANA (CARdiovascular risk in patients with DIAbetes in Navarra) cohort in 2016. To do so, a longitudinal extraction from multiple health and administrative databases of all patients in Navarra with Type 1 (T1D) and Type 2 (T2D) diabetes was conducted under the Real Word Data (RWD) framework. The baseline and first 5-year follow-up data collection ended in 2017. The aims of

For peer review only - http://bmjopen.bmj.com/site/about/guidelines.xhtml

setting up the CARDIANA cohort were: i) to establish a population-level dynamic cohort extraction and data integration mechanism that was nonexistent to date and could be used for research; ii) to assess which patient-level factors were determinant in the course of the disease in T1D and T2D patients of all ages, with a particular focus on socioeconomic factors; iii) to externally validate cardiovascular risk prediction models; iv) to assess if the inclusion of socioeconomic indicators on these models improves prediction performance; and v) to quantify the impact of health care provider and health care system actions on the CVD risk of this population.

Cohort description

The CARDIANA cohort is a population-based cohort from Navarra, an autonomous community located in northern region of Spain with approximately 650000 inhabitants and with a public health coverage (including both public and mixed coverage) over 99%.[8] It was designed by a multidisciplinary team involving methodologist, primary care specialist, endocrinologist, health care policy makers and clinical and social science researchers, many of them involved in the design of strategies for the management of patients with diabetes. The creation of this cohort was used as a 'case study' in the development of BARDENA, the Results Analysis Database of Navarra that is being constructed under the adoption of the Observational Medical Outcomes Partnership (OMOP) Common Data Model, which aims at harmonizing electronic medical records to facilitate participation on international distributed research.

The cohort includes all users of the Public Health Service of Navarra who, as of January 1st, 2012, had active codes of type 1 or type 2 diabetes (T89 and T90 of the International Classification of Primary Care, version 2, ICPC-2, respectively) in the Primary Care Electronic Medical Record System of Navarra (ATENEA) records. Patients with descriptions of diabetes different from T1D or T2D were excluded, as well as when severe inconsistencies in the dates of diagnosis, birth, or death were

For peer review only - http://bmjopen.bmj.com/site/about/guidelines.xhtml

found. Patients were also excluded if no registry of contact with the public health system was found either before the inclusion date and/or in the follow-up period. No other exclusion criteria were applied, and patients of all ages and conditions were considered, including T2D patients with onset during childhood and T1D patients with late onset during adulthood. Causes of early termination of the patient data extraction were death or change of community/country.

Figure 1 shows the flowchart of the creation of the cohorts. The classification of patients into T1D or T2D took into account that the validity of the T2D diagnosis had been assessed in a previous study,[9] but not that of the T1D diagnosis. Hence, the ICPC-2 codes and the descriptive field that goes with the code were first used, and after that, the classification procedure was complemented with the regional registry of T1D diabetes, which was legally approved by formal order 37/2014, on April 16th,[10] that includes all T1D patients with an onset date after 1989. More precisely, we first included all patients with ICPC-2 code T89 and T90 with active Individual Health Card in ATENEA. Second, we excluded patients with incompatible or incongruent information, such as to have died before 2012, to have unrealistic birth or onset dates, or to have diagnostic literals 'gestational diabetes' or 'pancreoprivic diabetes'. Third, using the regional registry of T1D diabetes, we maintained in the T2D cohort only patients that were not in the T1D registry, and passed all patients with T2D code to the T1D cohort if they were included in the T1D registry. Fourth, we excluded from the T1D cohort all patients that had onset date >1990 but were not in the T1D registry, and also all patients with onset date <1990 that were not treated with insulin. Finally, we excluded patients that had no contact with the health system before 2012, and patients that had no contact with the health system from 2012 to 2016. Combining the information from the health electronic records and administrative population datasets, two cohorts of prevalent patients with diabetes were created: the T1D CARDIANA cohort, with 1067 patients, and the T2D CARDIANA cohort, with 33842 patients. During the follow-up of the first wave, 33 (3.1%) T1D patients and 455 (1.3%) T2D patients were lost

For peer review only - http://bmjopen.bmj.com/site/about/guidelines.xhtml

to follow-up because of having moved to another region, and information for these patients was censored at this date accordingly.

The actual follow-up period of the cohort is five years, from January 1st 2012, to December 31st, 2016. The next data extraction process that will update longitudinal data and principal cardiovascular events will be conducted in the autumn of 2022, covering the period from January 1st 2017, to December 31st, 2021, and further extractions are planned in 5 year waves.

Variables, databases, and integration process

Sociodemographic and clinical variables of the defined CARDIANA T1D and CARDIANA T2D cohorts came from eight clinical and administrative databases: ATENEA, LAKORA-TIS, LAMIA, HCI, HIS-LEIRE (including the Minimum Basic Data Set at hospital discharge - MBDS), the population registry, the mortality registry, and the Type 1 diabetes registry. For future updates of the cohort, the Results Analysis Database of Navarra (BARDENA) will be used. A brief description of the original databases, which were extensively described elsewere,[11] is given in Supplementary Table S1, whereas a summary of the variables considered is given in Supplementary Table S2. In all, the information collected consists of all relevant structured data available from these sources generated during each contact of the patient with the health system. One set of variables was collected once and was considered fixed during the follow-up. These include the date of entry and/or exit from the health system, demographic and socioeconomic data such as the study level, lifestyle information such as tobacco use or physical activity level, the basic health zone the patients belongs to, coinsurance status[12], baseline comorbidities and past history of cardiovascular history using the ICPC-2 codification system, among others. Some new variables were created from these previous variables, such as the Charlson weighted score[13] or the GMA[14,15] comorbidity score. The other set of variables was collected longitudinally using a time-dependent structure and included all analytic results that

For peer review only - http://bmjopen.bmj.com/site/about/guidelines.xhtml

occurred during follow-up as well as pharmacologic treatments, health service use and fatal and nonfatal cardiovascular events. For these time-dependent variables, the date on which they occurred was also included. Cardiovascular events were considered to occur during the follow-up when CVD diagnostic codes were recorded in the mortality or the MBDS dataset, adapted from the codes of the EPIC-NL cohort given in Berkelmans et al.[16] (see the codes in Supplementary Table S2).

The integration procedure was conducted by the Statistic Institute of Navarra (NASTAT) and the Directorate-General for Informatics, Telecommunications, and Innovation of the Health Department of Navarra, who supervised the data extraction and guaranteed fulfillment of the law in terms of personal data protection. Afterward, the anonymized databases were provided to the research team.

Findings to date

The T1D and T2D CARDIANA cohorts consisted of 1067 and 33842 patients, respectively. Their sociodemographic characteristics are given in Table 1. No adjustment has been included due to the descriptive nature of the objective, but information on both cohorts is presented in parallel. Patients in both cohorts were primarily men (57.4% and 55.7% in T1D and T2D cohorts respectively), and only 5% were immigrants. Compared to patients in the T2D cohort, patients in the T1D cohort were much younger (mean age 36.9 years in T1D vs. 69.4 years in T2D), had a higher probability of being part of the workforce (84.5% vs. 26.6%), had a higher income level (38.3% vs. 27.5% had over 18,000 € per year) and also higher educational attainment (17.8% vs. 4.7% had university studies).

Health related patients' status at baseline, including lifestyle data, laboratory tests values, and office measured parameters and comorbidities are given in Table 2. The mean duration of diabetes was three years higher in T1D patients than in T2D patients (11.0 vs. 8.1 years), but their comorbidity indices were lower, with a weighted Charlson score equal to 1.2 vs. 2.1 and a weighted GMA equal

For peer review only - http://bmjopen.bmj.com/site/about/guidelines.xhtml

to 6.0 vs. 11.4, respectively. Similarly, T1D patients (4.9% vs. 23.8%) have much lower prevalence of cardiovascular disease history (4.9% vs. 23.8%), are more frequently active (71.5% vs 55.9%) and alcohol abstinent (69.0% vs 66.5%), but have higher probability of being smokers (32.2% vs 17.7%).

Regarding clinical and laboratory test parameters, T1D patients showed much better control of their body mass index (25.7 vs. 30.6 kg/m^2) but worse glycemic control of their HbA1c levels (8.2% vs. 7.1%). They also had better control of the other metabolic parameters considered, namely, high-density lipoprotein (62.0 vs. 48.9 mg/dL), low-density lipoprotein (105.7 vs. 111.4 mg/dL), triglycerides (84.2 vs. 142.7 mg/dL) and albumin to creatinine ratio levels (13.4 vs. 35.9).

The use of primary care services during the last year before baseline (Table 3) was lower in T1D than in T2D patients for the total number of visits (17.1 vs. 21.1 visits/year) and for all types of visits, except for emergency visits (0.6 vs. 0.5 visits/year) and remote visits to nursing (4.1 vs. 1.1 visits/year). Active prescriptions of antihypertensive medication were 41 points less frequent in patients with T1D (15.2% vs. 56.2%), and their lipid-lowering treatment use was less than half (18.8% vs. 49.3%). Similar differences were observed in antithrombotic treatment prescription (15.6% vs 38.2%), but baseline glucose-lowering treatment prescription was higher in patients with T1D (83.6% vs. 67.2%).

Only 17 patients (1.6%; 95% CI [1.0, 2.5]) in the T1D cohort developed a CVD event, five of which were fatal (0.46% of the total cohort). In the same follow up period, 14 patients died from non-cardiovascular related events. For the T2D cohort, 2882 (8.5%; 95% CI [8.2, 8.8]) had an event and 708 of them were fatal (2.1% of the total cohort). During this follow up, 5632 patients died from non-cardiovascular related events.

For the T2D cohort, the occurrence of CVD events along the follow-up has been associated with physical activity, with estimated ORs after matching and adjusting for confounders equal to 0.84

For peer review only - http://bmjopen.bmj.com/site/about/guidelines.xhtml

(95% CI: 0.66-1.07) for the partially active group and 0.71 (95% CI: 0.56-0.91) for the active group, compared with patients in the non-active group.[17]

Strengths and limitations of this study'

The main strength of this study is that the CARDIANA cohorts integrate exhaustive clinical, socioeconomic and behavioral information from all available administrative and clinical data sources, providing a complete framework to assess the course of the disease in patients with diabetes and the factors that affect it. Especially in relation to socioeconomic variables, these cohorts have individual information on country of origin, working status, educational level and income, which are not frequently available, with most studies using area-level proxies. Another strength is that data have been subjected to quality control procedures before and after database integration.

The main limitation of this study is the possible presence of bias resulting from the use of existing electronic clinical records, which may affect different methodological aspects. First, T2D patients without a diabetes code in the ATENEA records were not included. Although the validity of the code has been satisfactory asssessed,[9] undiagnosed patients have not been included, which in Spain it has been estimated that could account for 4-6% of the overall prevalence of 11-14%.[18,19] Second, data completeness can be low for some variables dependent on physicians' idiosyncratic reporting procedures, such as tobacco use or physical activity, and some variables that have been considered fixed may have changed along the follow-up. Although an effort has been made to complement variables with others that had a text format, information bias may be present, so imputation methods and sensitivity analyses will be required. Third, electronic prescriptions were only fully implemented in 2014, and at baseline it is estimated that 8-10% of the total prescriptions have not been accounted for. Fourth, patients without any contact with the regional public health system

For peer review only - http://bmjopen.bmj.com/site/about/guidelines.xhtml

because of using exclusively private health institutions were not included. Nevertheless, it is estimated that these patients account for less than 1% in the region.[8]

Collaboration

Requests for collaborative studies are welcome, upon request with a description of the planned projects from berta.ibanez.beroiz@navarra.es. They will only be considered after the approval of the research ethics committee from the solicitor institution and from the Navarra health system – Osasunbidea and the NASTAT institutions – responsible for the clinical information and the population information.

Patient and Public Involvement

None.

Ethics approval

The study protocol was approved by the Ethics Committee of Clinical Research of Navarra (Project 2015/111). This Committee approved, on August 19th 2022, to update the time-window of the cohorts to December 31st, 2021. This study has a retrospective nature, and data were irreversibly anonymized prior to transfer to the research team. The study was conducted according to the amended Declaration of Helsinki, Organic Law 3/2018, the General Data Protection Regulation (EU) 2016/679 and International Guidelines for Ethical Review of Epidemiological Studies.

Author contributions

BI and KC designed the study, researched the data and reviewed the manuscript. IT analyzed the data and wrote the manuscript. JLi, AG and ME researched the data and reviewed the manuscript. LF and MJG participated in the design of the study, in the acquisition of the T1D registry data and reviewed the manuscript. OL, JG, and AO participated in the acquisition and validation of the

For peer review only - http://bmjopen.bmj.com/site/about/guidelines.xhtml

For peer review only

ATENEA, LAKORA, LAMIA, HIS-Leire and HCI datasets and reviewed the manuscript. LA participated in the acquisition and validation of the population registry and reviewed the manuscript. CM participated in the acquisition and validation of the mortality registry database and reviewed the manuscript. JLa participated in the design of the study, contributed to the interpretation of the results and reviewed the manuscript. All authors have approved this version of the manuscript.

Funding

This work has received funding from the Instituto de Salud Carlos III through grant PI15/02196 and via the CONCEPT project (grants PI19/00154, PI19/00381). It has also received funding from REDISSEC RD16/0001/0014 and RICAPPS RD21/0016/0016, two Spanish Networks supported by Carlos III Health Institute and the European Regional Development Funding (FEDER). All funds are public and had no role in the design of the study, analysis or writing of the paper.

Data availability statement

Proposals for collaborative studies to share information on data are welcome, upon request with a description of the planned projects from berta.ibanez.beroiz@navarra.es. They will only be considered after the approval of the research ethics committee from the solicitor institution and from the Navarra health system – Osasunbidea and the NASTAT institutions – responsible for the clinical information and the population information.

Acknowledgements

The authors thank all professionals who participated in the acquisition of the data, especially Javier Baquedano, Adriana Rivero, Mª Ángeles Nuin and Alberto Jiménez-Idiazábal. The authors belong to the group RD16/0001/0014 of REDISSEC and to the group RD21/0016/0016 of RICAPPS.

For peer review only - http://bmjopen.bmj.com/site/about/guidelines.xhtml

For peer review only

Conflict of interest

None declared.

Figures

Figure 1: Type 1 and type 2 diabetes prevalent cohort creation flowchart

References

1. International Diabetes Federation. IDF Diabetes Atlas, 10th edition. 2022. Available from: https://www.diabetesatlas.org

2. The Lancet. Turning evidence into action on diabetes. *Lancet* [Internet]. Elsevier Ltd; 2020;**396**(10262):1535. Available from: http://dx.doi.org/10.1016/S0140-6736(20)32412-0

3. Peters SAE, Huxley RR, Sattar N, Woodward M. Sex Differences in the Excess Risk of Cardiovascular Diseases Associated with Type 2 Diabetes: Potential Explanations and Clinical Implications. *Curr Cardiovasc Risk Rep*. 2015;**9**(7):1–7.

4. Livingstone SJ, Looker HC, Hothersall EJ, et al. Risk of Cardiovascular Disease and Total Mortality in Adults with Type 1 Diabetes: Scottish Registry Linkage Study. *PLoS Med*. 2012;**9**(10).

5. Forbes JM, Cooper ME. Mechanisms of diabetic complications. *Physiol Rev*. 2013;**93**(1):137–188.

6. Galbete A, Tamayo I, Librero J, Enguita-Germán M, Cambra K, Ibáñez-Beroiz B. Cardiovascular risk in patients with type 2 diabetes: A systematic review of prediction models. *Diabetes Res Clin Pract*. 2022;**184**.

For peer review only - http://bmjopen.bmj.com/site/about/guidelines.xhtml

7. Ibáñez B, Galbete A, Goñi MJ, et al. Socioeconomic inequalities in cardiometabolic control in patients with type 2 diabetes. *BMC Public Health*. BMC Public Health; 2018;**18**(1):1–9.

8. Asistencia sanitaria. Cifras relativas. Instituto Nacional Estadística (INE), 2018. https://www.ine.es/jaxi/Tabla.htm?tpx=47935&L=0.

9. Moreno-Iribas C, Sayon-Orea C, Delfrade J, et al. Validity of type 2 diabetes diagnosis in a population-based electronic health record database. *BMC Med Inform Decis Mak*. BMC Medical Informatics and Decision Making; 2017;**17**(1):1–6.

10. Orden Foral 37/2014. Navarra,Spain: https://bon.navarra.es/es/anuncio/-/texto/2014/88/10; 2014.

11. Moulis G, Ibañez B, Palmaro A, et al. Cross-national health care database utilization between spain and france: results from the epichronic study assessing the prevalence of type 2 diabetes mellitus. *Clin Epidemiol*. 2018;**10**:863–874.

12. Pozo-Rubio R del, Pardo-García I, Escribano-Sotos F. El copago de dependencia en España a partir de la reforma estructural de 2012. *Gac Sanit*. 2017;**31**(1):23–29.

13. Charlson ME, Pompei P, Ales KL, MacKenzie CR. A new method of classifying prognostic comorbidity in longitudinal studies: development and validation. *J Chronic Dis*. England; 1987;**40**(5):373–383.

14. González González AIG, Miquel Gómez AM, Rodríguez Morales D, et al. Concordancia y utilidad de un sistema de estratificación para la toma de decisiones clínicas. *Atención Primaria*. 2017;**49**(4):240–247.

15. Monterde D, Vela E, Clèries M. Adjusted morbidity groups: A new multiple morbidity

For peer review only - http://bmjopen.bmj.com/site/about/guidelines.xhtml

measurement of use in Primary Care. *Aten Primaria* [Internet]. Elsevier España, S.L.U.; 2016;**48**(10):674–682. Available from: http://dx.doi.org/10.1016/j.aprim.2016.06.003

16. Berkelmans GFN, Gudbjörnsdottir S, Visseren FLJ, Wild SH, Franzen S, Chalmers J, Davis BR, Poulter NR, Spijkerman AM, Woodward M, Pressel SL, Gupta AK, van der Schouw YT, Svensson AM, van der Graaf Y, Read SH, Eliasson B, Dorresteijn JAN. Prediction of individual life-years gained without cardiovascular events from lipid, blood pressure, glucose, and aspirin treatment based on data of more than 500 000 patients with Type 2 diabetes mellitus. Eur Heart J. 2019 Sep 7;40(34):2899-2906.

17. Enguita-Germán M, Tamayo I, Galbete A, Librero J, Cambra K, Ibáñez-Beroiz B. Effect of physical activity on cardiovascular event risk in a population-based cohort of patients with type 2 diabetes. *Int J Environ Res Public Health*. 2021;**18**(23).

18. Soriguer F, Goday A, Bosch-Comas A, et al. Prevalence of diabetes mellitus and impaired glucose regulation in Spain: The Di@bet.es Study. *Diabetologia*. 2012;**55**(1):88–93.

19. Aguayo A, Urrutia I, González-Frutos T, et al. Prevalence of diabetes mellitus and impaired glucose metabolism in the adult population of the Basque Country, Spain. *Diabet Med*. 2017;**34**(5):662–666.

For peer review only - http://bmjopen.bmj.com/site/about/guidelines.xhtml

Table 1: Demographic and socioeconomic characteristics of the T1D and T2DCARDIANA cohorts at baseline (January 1st, 2012)

	T1D CARDIANA Cohort			T2D CARDIANA Cohort		
	Male	Female	**Total**	Male	Female	**Total**
**n**	612	455	1067	18840	15002	33842
**Age**, Mean (SD)	36.6 (15.9)	37.2 (17.8)	36.9 (16.7)	67.1 (12.3)	72.3 (13.0)	69.4 (12.8)
**Working status, n(%)**						
Unemployed	36 (7.8)	21 (6.7)	57 (7.3)	836 (5.0)	609 (5.8)	1445 (5.3)
Working	392 (84.5)	264 (84.6)	656 (84.5)	5506 (33.1)	1713 (16.3)	7219 (26.6)
Pensioner	36 (7.8)	27 (8.7)	63 (8.1)	10308 (61.9)	8157 (77.8)	18465 (68.1)
(Missing)	*148*	*143*	*291*	*2190*	*4523*	*6713*
**Continent of origin, n(%)**						
Spain	559 (94.6)	415 (94.7)	974 (94.7)	17645 (95.9)	13853 (94.7)	31498 (95.3)
Europe	15 (2.5)	5 (1.1)	20 (1.9)	293 (1.6)	240 (1.6)	533 (1.6)
Africa	9 (1.5)	6 (1.4)	15 (1.5)	171 (0.9)	115 (0.8)	286 (0.9)
America	8 (1.4)	11 (2.5)	19 (1.8)	269 (1.5)	399 (2.7)	668 (2.0)
Asia	0 (0.0)	1 (0.2)	1 (0.1)	23 (0.1)	29 (0.2)	52 (0.2)
Australia	0 (0.0)	0 (0.0)	0 (0.0)	1 (0.0)	0 (0.0)	1 (0.0)
(Missing)	*21*	*17*	*38*	*438*	*366*	*804*
**Copayment category, n(%)**						
<18000	338 (56.4)	301 (68.9)	639 (61.7)	11445 (62.9)	12223 (84.6)	23668 (72.5)
≥18000	261 (43.6)	136 (31.1)	397 (38.3)	6747 (37.1)	2229 (15.4)	8976 (27.5)
(Missing)	13	18	31	648	550	1198
**Study level, n(%)**						
No formal education	132 (22.8)	79 (18.5)	211 (21.0)	5245 (28.6)	6033 (41.4)	11278 (34.3)
Primary School	226 (39.1)	157 (36.9)	383 (38.1)	9744 (53.1)	7410 (50.8)	17154 (52.1)
High School	136 (23.5)	95 (22.3)	231 (23.0)	2227 (12.1)	711 (4.9)	2938 (8.9)
University level	84 (14.5)	95 (22.3)	179 (17.8)	1121 (6.1)	424 (2.9)	1545 (4.7)
(Missing)	*34*	*29*	*63*	*503*	*424*	*927*
**Mean income, Mean (SD)**	12011.6 (1803.8)	12099.3 (1742.3)	12048.9 (1777.6)	11748.2 (1845.8)	11531.6 (1739.5)	11652.3 (1802.7)
**Income Quintile, n(%)**						
[7300,10565)	128 (21.7)	78 (17.8)	206 (20.0)	3539 (19.2)	3144 (21.5)	6683 (20.2)
[10565,11416)	110 (18.6)	97 (22.1)	207 (20.1)	3515 (19.1)	3099 (21.2)	6614 (20.0)
[11416,12240)	122 (20.6)	84 (19.2)	206 (20.0)	3577 (19.4)	2980 (20.4)	6557 (19.8)
[12240,13394)	118 (20.0)	88 (20.1)	206 (20.0)	3820 (20.8)	2799 (19.1)	6619 (20.0)
[13394,17708]	113 (19.1)	91 (20.8)	204 (19.8)	3949 (21.5)	2615 (17.9)	6564 (19.9)
(Missing)	*21*	*17*	*38*	*440*	*365*	*805*

Percentage for each category are column percentages (% of patients in each category for each cohort), unless otherwise indicated (mean and standard deviations- SD are given in quantitative variables)

Table 2: Clinical and lifestyle characteristics of the T1D and T2D CARDIANA cohorts at baseline (January 1st, 2012)

		T1D CARDIANA Cohort			T2D CARDIANA Cohort		
		Male	Female	**Total**	Male	Female	**Total**
**n**		612	455	1067	18840	15002	33842
Clinical parameters, mean (SD)							
Duration of diabetes (years)		10.8 (9.2)	11.1 (9.1)	11.0 (9.1)	7.8 (5.8)	8.5 (6.3)	8.1 (6.0)
Body Mass Index (Kg/m2)		26.2 (4.5)	25.2 (5.0)	25.7 (4.7)	30.2 (5.3)	31.0 (6.4)	30.6 (5.8)
Systolic Blood Pressure (mm Hg)		124.0(19.0)	121.4(19.9)	122.9(19.4)	135.5 (16.9)	135.7 (17.9)	135.6 (17.3)
Diastolic Blood Pressure (mm Hg)		72.3 (12.3)	71.0 (9.8)	71.7 (11.2)	76.6 (10.5)	75.8 (10.5)	76.2 (10.5)
Laboratory tests, mean (SD)							
HbA1c (%)		8.2 (1.6)	8.2 (1.4)	8.2 (1.5)	7.0 (1.3)	7.1 (1.3)	7.1 (1.3)
Fasting glucose (mg/dL)		179.9(97.6)	172(80.5)	176.3(89.3)	141.6 (44.6)	139.0 (45.4)	140.4 (45.0)
Total Cholesterol (mg/dL)		188(42.1)	188(31.2)	188(36.9)	183.2 (39.2)	194.0 (37.9)	188.0 (39.0)
High Density Lipoprotein (mg/dL)		57.5 (17.0)	68.4 (17.8)	62.0 (18.2)	46.3 (13.3)	52.1 (14.4)	48.9 (14.1)
Low Density Lipoprotein (mg/dL)		107.2(29.8)	103.6(28.7)	105.7(29.4)	108.9 (31.9)	114.4 (32.4)	111.4 (32.2)
Triglicerides (mg/dL)		90.7 (58.5)	75.1 (37.7)	84.2 (51.4)	141.5 (77.3)	144.1 (68.9)	142.7 (73.7)
Creatinine (mg/dL)		0.9 (0.3)	0.7 (0.2)	0.8 (0.3)	1.1 (0.6)	0.9 (0.5)	1.0 (0.6)
Albumin to creatinine ratio		12.9 (30.9)	14.0 (31.5)	13.4 (31.2)	40.2 (165.6)	30.4 (134.0)	35.9 (152.4)
Lifestyle data, n(%)							
Smoking Status	Non Smoker	149 (49.2)	150 (60.2)	299 (54.2)	6009 (37.9)	11025(85.1)	17034(59.1)
	Ex-smoker	51 (16.8)	24 (9.6)	75 (13.6)	5810 (36.6)	887 (6.8)	6697 (23.2)
	Smoker	103 (34.0)	75 (30.1)	178 (32.2)	4052 (25.5)	1041 (8.0)	5093 (17.7)
	(Missing)	*309*	*206*	*515*	*2969*	*2049*	*5018*
Alcohol	Abstinent	150 (59.5)	159 (81.1)	309 (69.0)	7082 (47.5)	10805(90.1)	17887(66.5)
	Moderate drinker	95 (37.7)	37 (18.9)	132 (29.5)	7044 (47.3)	1137 (9.5)	8181 (30.4)
	Heavy drinker	7 (2.8)	0 (0.0)	7 (1.6)	777 (5.2)	44 (0.4)	821 (3.1)
	(Missing)	*360*	*259*	*619*	*3937*	*3016*	*6953*
Physical activity	Inactive	7 (4.0)	11 (7.6)	18 (5.6)	1431 (9.8)	1931 (16.1)	3362 (12.6)
	Partially active	38 (21.7)	35 (24.3)	73 (22.9)	3966 (27.2)	4405 (36.8)	8371 (31.5)
	Active	130 (74.3)	98 (68.1)	228 (71.5)	9207 (63.0)	5647(47.1)	14854(55.9)
	(Missing)	*437*	*311*	*748*	*4236*	*3019*	*7255*
Comorbidities							
Charlson score, mean (SD)		1.2 (0.8)	1.2 (0.8)	1.2 (0.8)	2.2 (1.7)	2.1 (1.6)	2.1 (1.7)
GMA[a] score, mean (SD)		5.5 (3.8)	6.7 (4.7)	6.0 (4.2)	10.7 (5.8)	12.2 (5.9)	11.4 (5.9)
Previous CVD		31 (5.1)	21 (4.6)	52 (4.9)	4804 (25.5)	3236 (21.6)	8040 (23.8)
Diabetes related comorbidities, n(%)							
Retinopathy (%)	Yes	25 (35.2)	11 (18.6)	36 (27.7)	670 (15.3)	616 (18.9)	1286 (16.8)
	(Missing)	*541*	*396*	*937*	*14451*	*11738*	*26189*
Amputation	Yes	1 (1.2)	0 (0.0)	1 (0.7)	160 (2.0)	54 (0.8)	214 (1.5)
	(Missing)	*532*	*390*	*922*	*10865*	*8252*	*19117*
Diabetic foot risk	None	4 (5.9)	4 (6.2)	8 (6.0)	230 (3.2)	212 (3.5)	442 (3.3)
	Superficial ulcer	53 (77.9)	56 (86.2)	109 (82.0)	6048 (83.6)	4809 (79.7)	10857(81.8)
	Deep tissue ulcers w/o abcess	8 (11.8)	3 (4.6)	11 (8.3)	652 (9.0)	704 (11.7)	1356 (10.2)
	Deep tissue ulcers w abcess	2 (2.9)	1 (1.5)	3 (2.3)	182 (2.5)	244 (4.0)	426 (3.2)
	Localized gangrene	1 (1.5)	1 (1.5)	2 (1.5)	113 (1.6)	62 (1.0)	175 (1.3)
	Extensive gangrene	0 (0.0)	0 (0.0)	0 (0.0)	10 (0.1)	4 (0.1)	14 (0.1)
	(Missing)	*544*	*390*	*934*	*11605*	*8967*	*20572*
Cardiovascular risk scores, mean (SD)							
SCORE[b], mean (SD)		1.9 (2.6)	0.8 (1.2)	1.4 (2.1)	4.9 (6.6)	2.0 (1.7)	3.6 (5.3)

Percentage for each category are column percentages (% of patients in each category for each cohort) unless otherwise indicated (mean and standard deviations- SD are given in quantitative variables)

[a]GMA: Adjusted Morbidity groups ;[b]SCORE: Systematic COronary Risk Evaluation

For peer review only - http://bmjopen.bmj.com/site/about/guidelines.xhtml

Table 3: Use of Primary Care Services during the year previous to baseline (2011) and active prescriptions at baseline (January 1st, 2012)

	T1D CARDIANA Cohort			T2D CARDIANA Cohort		
	Male	Female	**Total**	Male	Female	**Total**
**n**	612	455	1067	18840	15002	33842
**Total visits,** mean (SD)	15.9 (11.4)	19.0 (12.6)	17.1 (12.2)	19.4 (19.1)	23.2 (20.8)	21.1 (19.9)
**Visits at Office, by professional,** mean(SD)						
Nursing	5.2 (6.7)	5.6 (6.9)	5.4 (6.8)	7.1 (10.3)	7.6 (9.7)	7.3 (10.1)
Physician	3.8 (4.3)	5.2 (5.5)	4.4 (4.9)	6.4 (5.8)	7.3 (6.5)	6.8 (6.1)
Social Worker	0.8 (1.4)	1.3 (3.4)	1.0 (2.5)	1.7 (2.4)	1.9 (3.6)	1.7 (3.0)
Emergency	0.1 (0.3)	0.1 (0.5)	0.1 (0.4)	0.1 (0.6)	0.2 (0.9)	0.1 (0.8)
Other	0.6 (1.7)	0.7 (1.5)	0.6 (1.6)	0.5 (2.7)	0.6 (1.8)	0.5 (2.3)
**Visits at home, by professional,** mean(SD)						
Nursing	0.1 (0.8)	0.0 (0.4)	0.1 (0.7)	0.7 (5.7)	1.4 (7.8)	1.0 (6.7)
Physician	0.1 (0.3)	0.0 (0.2)	0.0 (0.3)	0.3 (1.7)	0.6 (2.2)	0.4 (1.9)
Social Worker	0.0 (0.1)	0.0 (0.2)	0.0 (0.2)	0.1 (1.2)	0.2 (1.6)	0.1 (1.4)
Emergency	0.0 (0.0)	0.0 (0.1)	0.0 (0.0)	0.0 (0.1)	0.0 (0.2)	0.0 (0.2)
Other	0.0 (0.3)	0.0 (0.1)	0.0 (0.3)	0.1 (0.6)	0.1 (0.8)	0.1 (0.7)
**Remote visits, by professional,** mean(SD)						
Nursing	3.9 (4.9)	4.3 (5.6)	4.1 (5.2)	0.9 (2.3)	1.3 (2.8)	1.1 (2.6)
Physician	1.4 (2.3)	1.8 (2.9)	1.5 (2.6)	1.5 (3.1)	2.0 (3.9)	1.7 (3.5)
Social Worker	0.0 (0.1)	0.0 (0.1)	0.0 (0.1)	0.0 (0.2)	0.0 (0.2)	0.0 (0.2)
Emergency	0.0 (0.2)	0.0 (0.2)	0.0 (0.2)	0.0 (0.4)	0.1 (0.6)	0.1 (0.5)
**Drug treatments use,** n(%)						
Antihypertensive	95 (15.5)	67 (14.7)	162 (15.2)	10303(54.7)	8769(58.5)	19072(56.4)
Glucose-lowering	519 (84.8)	373 (82.0)	892 (83.6)	12630(67.0)	10099(67.3)	22729(67.2)
Lipid-lowering	122 (19.9)	79 (17.4)	201 (18.8)	9423(50.0)	7249(48.3)	16672(49.3)
Antithrombotic	67 (10.9)	47 (10.3)	114 (10.7)	7722(41.0)	5262(35.1)	12984(38.4)

Percentage for each category are row percentages (% of patients with each treatment in each cohort), unless otherwise indicated (mean and standard deviations- SD are given in quantitative variables)

For peer review only - http://bmjopen.bmj.com/site/about/guidelines.xhtml

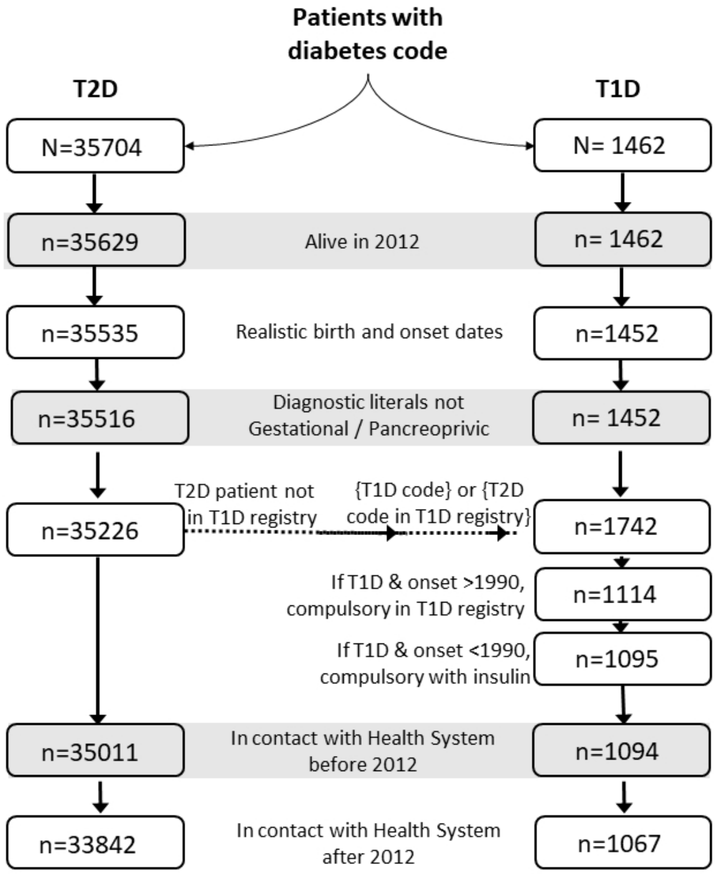

Figure 1: Type 1 and type 2 diabetes prevalent cohort creation flowchart

190x275mm (96 x 96 DPI)

Supplementary Table S1: Original Data Sources used for the creation of the T1D and T2D cohorts$^{\Omega}$

ATENEA –The Primary Care Electronic Medical Record System of Navarra
What contains: out-hospital data. Includes demographic information, visits to primary care services (data and type), health problems, life style, detailed clinical data, laboratory results and drug prescription data.<br>*Code system used:* ICPC-2 International Classification of Primary Care, version 2<br>*Implemented:* in 2003, with a 100% of use in 2008
LAKORA – Population Information System (within the Health system)
What contains: Basic information on coverage, insurance modality, pharmaceutical copayment status, primary healthcare district, country of origin and other administrative data.<br>*Code system used*: free text<br>*Implemented: in 2003,* with a 100% of use in 2008
MORTALITY Registry
What contains: Date and cause of death<br>*Code system used*: International Classification of Diseases ICD-10-ES
HIS-LEIRE (with the Minimum Basic Data Set, MBDS)
What contains: It contains the MBDS, with socio-demographic and clinical information on all hospital discharges and major ambulatory surgery in the hospitals of Navarra, including partnership hospitals. It also contains information about visits to specialist or to day hospitals<br>*Code system used*: International Classification of Diseases; ICD9CM until December, 2015 and ICD-10-ES from January, 2016 on.<br>*Implemented*: The MBDS was compulsory in Spain since 1992. HIS-LEIRE had 100% of use in 2003
HCI- Clinical data from specialized care
What contains: comprehensive information of specialized care, outpatient consultation, lab test, images, visits and other data from specialized care.<br>*Code system used*: free text<br>*Implemented*: In 2001, with 100% of use in 2008
T1D registry
What contains: demographic and clinical information of all patients with T1D diabetes to estimate incidence, prevalence, morbidity and mortality of T1D patients<br>*Implemented*: in 2014, legally approved by formal order 37/2014
Population registry
What contains: At individual level: study level, nationality, labor force status (employed, unemployed, pensioner); at area-level (census tract): average income (2013), % unemployed, %immigration, % people without studies<br>*Code system used*: For study level: Normalized Classification of Education (CNE) used for census in the INE (National Institute of Statistics)[£]
LAMIA
What contains: e-prescription paper-free system with both prescriptions and dispensations, connected to all community pharmacies in the region<br>*Code system used*: Anatomical Therapeutic Chemical (ATC) code<br>*Implemented*: In 2013, with 100% of use in 2014. Before 2014, information on prescriptions was obtained from ATENEA.

$^{\Omega}$ For future updates of the cohort, the Results Analysis Database of Navarre (BARDENA) will be used

[£] https://idapadron.ine.es/repositorio/legislacion/Anexo%20II.pdf

For peer review only

Supplementary Table S2: Data information available in the T1D and T2D cohorts, by type of information and data source

Data Source	Variables
**Socioeconomic data**	
Population Registry	Date of birth, country of origin, study level, marital status, working status and mean income of the assigned basic health zone
LAKORA-TIS	Pharmaceutical co-payment category
ATENEA-TIS	Date of birth, date of inclusion/withdrawal in the Navarra health system, general practitioner identification
**Previous diagnosis and past history of diseases**	
ATENEA	Weighted GMA, Past-history of myocardial infarction, congestive heart failure, cerebrovascular disease, peripheral vascular disease, retinopathy, neuropathy, nephropathy, foot lesions, dementia, chronic pulmonary disease, rheumatic disease, peptic ulcer disease, hemiplegia or paraplegia, neoplasm, liver disease, AIDS/HIV. Past history of CVD events coded in one of the following ICPC-2 codes: K74, K75, K76, K77, K89, K90, K91
**Personal cardiovascular risk factors, laboratory test and lifestyle behavior**	
ATENEA	Sex, age, physical activity, alcohol consumption, smoking status, self-management of pharmacological treatment, time since diagnosis, office, and laboratory test parameters (and dates): weight, height, systolic blood pressure (SBP), diastolic blood pressure (DBP), Total Cholesterol, LDL, HDL, triglycerides, creatinine, haemoglobin, glycated haemoglobin, fasting glucose, glomerular filtration rate (GFR), urine albumin to creatinine ratio.
The HCI database	Data collected by specialist care units: office and laboratory test parameters and procedures (date and value)
**Use of health services**	
HIS-LEIRE	Hospital or emergency room admissions, diagnostics, procedures and dates of admission and discharge.<br>Number of consultations to specialist by medical specialty
ATENEA	Number of yearly telephonic/physical consultation, office/home-based consultation and type of service provided (nurse/ medical/social worker/Emergency room)
**Drug treatment use**	
LAMIA	Treatment ATC code, date of prescription and date of dispensation
ATENEA	Treatment ATC code and date of prescription (before 2014)
**Diabetes classification**	
T1D registry	Type 1 diabetes confirmation and onset date
**Outcomes**	
Mortality registry	Cause of death and date
HIS-LEIRE/MBDS	Minimum Basic Data Set (MBDS): ICD-9CM until December 31st, 2015 and ICD-10-ES from January 1st, 2016.<br><br>Hospitalization or death related to a cardiovascular event coded in one of the following: ICD-9 codes: 410,430,431,432,433,434,435;ICD-10 codes: I20-I25, I46,I60-I63,I65,G45

BMJ Open

Cohort Profile: CArdiovascular Risk in patients with DIAbetes in Navarra (CARDIANA cohort)

Journal:	*BMJ Open*
Manuscript ID	bmjopen-2022-066052.R2
Article Type:	Cohort profile
Date Submitted by the Author:	28-Dec-2022

Complete List of Authors:	Tamayo, Ibai; Navarrabiomed, IdiSNA, Unidad de Metodología; Red de Investigación en Cronicidad, Atención Primaria y Promoción de la Salud<br>Librero-Lopez, Julian ; Navarrabiomed, IdiSNA; Red de Investigacion en Servicios de Salud en Enfermedades Cronicas, Red de Investigación en Cronicidad, Atención Primaria y Servicios de Salud<br>Galbete, Arkaitz; Universidad Pública de Navarra, Estadística; Navarrabiomed, IdISNA<br>Cambra, Koldo; Departamento de Sanidad. Gobierno Vasco.<br>Enguita, Mónica; Navarrabiomed, IdiSNA; Red de Investigación en Servicios de Salud en Enfermedades Crónicas, Red de Investigación en Cronicidad, Atención Primaria y Promoción de la Salud<br>Forga, Luis; Servicio Navarro de Salud - Osasunbidea, Servicio de Endocrinología y Nutrición - HUN; Red de Investigación en Servicios de Salud en Enfermedades Crónicas, Red de Investigación en Cronicidad, Atención Primaria y Promoción de la Salud<br>Goñi, José; Servicio Navarro de Salud - Osasunbidea, Servicio de Endocrinología y Nutrición - HUN; Red de Investigación en Servicios de Salud en Enfermedades Crónicas, Red de Investigación en Cronicidad, Atención Primaria y Promoción de la Salud<br>Lecea, Oscar; Servicio Navarro de Salud - Osasunbidea, Atención Primaria; Red de Investigación en Servicios de Salud en Enfermedades Crónicas, Red de Investigación en Cronicidad, Atención Primaria y Promoción de la Salud<br>Gorricho, Javier; Servicio Navarro de Salud - Osasunbidea, Servicio de Evaluación y Difusión de resultados en Salud; Red de Investigacion en Servicios de Salud en Enfermedades Cronicas, Red de Investigación en Cronicidad, Atención Primaria y Servicios Sanitarios<br>Olazarán, Álvaro; Departamento de Universidad, Innovación y Transformación, Servicio Tecnologías de la Salud<br>Arnedo, Laura; Instituto de Estadística de Navarra<br>Moreno-Iribas , Conchi; Instituto de Salud Pública y Laboral de Navarra; Red de Investigacion en Servicios de Salud en Enfermedades Cronicas, Red de Investigación en Cronicidad, Atención Primaria y Promoción de la Salud<br>Lafita, Javier; Servicio Navarro de Salud - Osasunbidea, Servicio de Efectividad y Seguridad Asistencial ; Red de Investigación en Servicios de Salud en Enfermedades Crónicas, Red de Investigación en Cronicidad, Atención Primaria y Promoción de la Salud<br>Ibañez, Berta; Navarrabiomed, IdiSNA; Red de Investigacion en Servicios de Salud en Enfermedades Cronicas, Red de Investigación en Cronicidad, Atención Primaria y Promoción de la Salud

**Primary Subject Heading**:	Epidemiology
Secondary Subject Heading:	Diabetes and endocrinology, Cardiovascular medicine
Keywords:	General diabetes < DIABETES & ENDOCRINOLOGY, Cardiac Epidemiology < CARDIOLOGY, Risk management < HEALTH SERVICES ADMINISTRATION & MANAGEMENT, Health informatics < BIOTECHNOLOGY & BIOINFORMATICS

SCHOLARONE™ Manuscripts

I, the Submitting Author has the right to grant and does grant on behalf of all authors of the Work (as defined in the below author licence), an exclusive licence and/or a non-exclusive licence for contributions from authors who are: i) UK Crown employees; ii) where BMJ has agreed a CC-BY licence shall apply, and/or iii) in accordance with the terms applicable for US Federal Government officers or employees acting as part of their official duties; on a worldwide, perpetual, irrevocable, royalty-free basis to BMJ Publishing Group Ltd ("BMJ") its licensees and where the relevant Journal is co-owned by BMJ to the co-owners of the Journal, to publish the Work in this journal and any other BMJ products and to exploit all rights, as set out in our licence.

The Submitting Author accepts and understands that any supply made under these terms is made by BMJ to the Submitting Author unless you are acting as an employee on behalf of your employer or a postgraduate student of an affiliated institution which is paying any applicable article publishing charge ("APC") for Open Access articles. Where the Submitting Author wishes to make the Work available on an Open Access basis (and intends to pay the relevant APC), the terms of reuse of such Open Access shall be governed by a Creative Commons licence – details of these licences and which Creative Commons licence will apply to this Work are set out in our licence referred to above.

Other than as permitted in any relevant BMJ Author's Self Archiving Policies, I confirm this Work has not been accepted for publication elsewhere, is not being considered for publication elsewhere and does not duplicate material already published. I confirm all authors consent to publication of this Work and authorise the granting of this licence.

For peer review only

Cohort Profile: CArdiovascular Risk in patients with DIAbetes in Navarra (CARDIANA cohort)

Ibai Tamayo[a,b] , Julián Librero[a,b], Arkaitz Galbete[a,c], Koldo Cambra[b,d], Mónica Enguita[a,b], Luis Forga[b,e], Mª José Goñi[b,e], Oscar Lecea[b,f], Javier Gorricho[b,g], Álvaro Olazarán[h], Laura Arnedo[i], Conchi Moreno-Iribas[b,j], Javier Lafita[b,k] and Berta Ibáñez-Beroiz*[a,b]

a. Unidad de Metodología. Navarrabiomed-Hospital Universitario de Navarra-Universidad Pública de Navarra, IdiSNA, Pamplona, Spain
b. Red de Investigación en Servicios Sanitarios y Enfermedades Crónicas (REDISSEC) y Red de Investigación en Cronicidad, Atención Primaria y Promoción de la Salud (RICAPPS), Pamplona, Spain
c. Departamento de Estadística. Universidad Pública de Navarra (UPNA), Pamplona, Spain
d. Dirección de Salud Pública y Adicciones, Departamento de Sanidad, Gobierno Vasco, Vitoria, Spain
e. Servicio de Endocrinología y Nutrición, Hospital Universitario de Navarra, Servicio Navarro de Salud (SNS-O), Pamplona, Spain
f. Atención Primaria, Servicio Navarro de Salud (SNS-O), Pamplona, Spain
g. Servicio de Evaluación y Difusión de resultados en Salud, Servicio Navarro de Salud (SNS-O), Pamplona, Spain
h. Servicio Tecnologías de la Salud, Dpto de Universidad, Innovación y Transformación, Pamplona, Spain
i. Instituto de Estadística de Navarra (NASTAT), Pamplona, Spain
j. Instituto de Salud Pública y Laboral de Navarra (ISPLN), Pamplona, Spain
k. Servicio de Efectividad y Seguridad Asistencial (SNS-O), Pamplona, Spain

For peer review only

For peer review only - http://bmjopen.bmj.com/site/about/guidelines.xhtml

ibai.tamayo.rodriguez@navarra.es

julian.librero.lopez@navarra.es

arkaitz.galbete@unavarra.es

k-cambra@euskadi.eus

monica.enguita.german@navarra.es

lluis.forga.llenas@navarra.es

mj.goni.iriarte@navarra.es

oscar.lecea.juarez@navarra.es

javier.gorricho.mendivil@navarra.es

alvaro.olazaran.santesteban@navarra.es

laura.arnedo.ajona@navarra.es

mc.moreno.iribas@navarra.es

javier.lafita.tejedor@navarra.es

Correspondence to:

Berta Ibáñez-Beroiz

Navarrabiomed-Complejo Hospitalario de Navarra-Universidad Pública de Navarra, C/ Irunlarrea s/n. Recinto CHN, 31008, Pamplona, Spain

berta.ibanez.beroiz@navarra.es

Word count: 2769

For peer review only - http://bmjopen.bmj.com/site/about/guidelines.xhtml

Abstract

Purpose: The CARDIANA cohort was established to assess the effects of sociodemographic and clinical variables on the risk of cardiovascular events in patients with type 1 (T1D) or type 2 (T2D) diabetes, with a special focus on socioeconomic factors, and to validate and develop cardiovascular risk models for these patients.

Participants: The CARDIANA cohort included all patients with T1D and T2D diabetes registered in the Public Health Service of Navarra with prevalent disease on January 1, 2012. It consisted of 1067 T1D patients (ages 2-88 years) and 33842 T2D patients (ages 20-105 years), whose data were retrospectively extracted from the Health and Administrative System Databases.

Findings to date: The follow-up period for Wave 1 was from January 1, 2012 to December 31, 2016. During these five years, 9 patients (0.8%; 95% CI [0.4, 1.6]) in the T1D cohort developed a CVD event, whereas for the T2D cohort, 2602 (7.7%; 95% CI [7.4, 8.0]) had an event. For the T2D cohort, physical activity was associated with a reduced risk of cardiovascular events, with adjusted estimated odds ratios equal to 0.84 (95% CI: 0.66-1.07) for the partially active group and 0.71 (95% CI: 0.56-0.91) for the active group, compared with patients in the non-active group.

Future plans: The CARDIANA cohort is currently being used to assess the effect of sociodemographic risk factors on CV risk at 5 years and to externally validate cardiovascular predictive models. A second Wave is being conducted in late 2022 and early 2023, to extend the follow-up other five years, from January 1, 2016 to December 31, 2021. Periodic data extractions are planned every five years.

For peer review only - http://bmjopen.bmj.com/site/about/guidelines.xhtml

Strengths and limitations of this study

- The CARDIANA cohorts integrate exhaustive clinical, socioeconomic and behavioral information from all available administrative and clinical data sources in patients with type 1 and type 2 diabetes.
- The data have been subject to quality control procedures before and after database integration.
- The presence of possible bias resulting from the use of existing electronic clinical records needs to be accounted for.
- Some variables may be underreported, and electronic prescriptions were only fully implemented in 2014.

For peer review only

Introduction

Diabetes mellitus is a common metabolic disorder that affected one in ten adults worldwide in 2021. Approximately 11.5% of the total health care spending and 12.2% of global all-cause deaths in adults aged 20-79 years are attributable to diabetes.[1] Despite governments agreeing to halt the increase in diabetes and obesity by 2025,[2] projections for 2045 show a growth of 16% in the expected prevalence of diabetes, becoming one of the fastest growing global health emergency of the 21st century.[1]

Patients with diabetes develop common macro- and microvascular complications that result in an increased cardiovascular disease (CVD) risk.[3] Stratification of patients with diabetes according to their CVD risk and proper management has become an essential need for health care providers. However, identifying which factors and interventions impact the course of the disease is not straightforward, because their impact can differ among cohorts depending on the socioeconomic context, on the health care provider practices and also because of the differences in the etiology of type 1 and type 2 diabetes.[4,5] Focusing on this need, several cardiovascular prediction models have been proposed over the years, some of them specifically designed for patients with diabetes.[6] Choosing the CVD risk model to be applied in a particular health system is not trivial, since external validations of the models are scarce and implementation procedures are rarely straightforward.

Taking advantage of the quality of the administrative and clinical datasets in Navarra, already used for research in patients with type 2 Diabetes,[7] we initiated the creation of the population-based CARDIANA (CARdiovascular risk in patients with DIAbetes in Navarra) cohort in 2016. To do so, a longitudinal extraction from multiple health and administrative databases of all patients in Navarra with Type 1 (T1D) and Type 2 (T2D) diabetes was conducted under the Real Word Data (RWD)

For peer review only - http://bmjopen.bmj.com/site/about/guidelines.xhtml

framework. The baseline and first 5-year follow-up data collection ended in 2017. The aims of setting up the CARDIANA cohort were: i) to establish a population-level dynamic cohort extraction and data integration mechanism that was nonexistent to date and could be used for research; ii) to assess which patient-level factors were determinant in the course of the disease in T1D and T2D patients of all ages, with a particular focus on socioeconomic factors; iii) to externally validate cardiovascular risk prediction models; iv) to assess if the inclusion of socioeconomic indicators on these models improves prediction performance; and v) to quantify the impact of health care provider and health care system actions on the CVD risk of this population.

Cohort description

The CARDIANA cohort is a population-based cohort from Navarra, an autonomous community located in a northern region of Spain with approximately 650 000 inhabitants and with a public health coverage (including both public and mixed coverage) over 99%.[8] It was designed by a multidisciplinary team involving methodologists, primary care specialists, endocrinologists, health care policy makers and clinical and social science researchers, many of them with expertise in the design of strategies for the management of patients with diabetes. The creation of this cohort was used as a 'case study' in the development of BARDENA, the Results Analysis Database of Navarra that is being constructed under the adoption of the Observational Medical Outcomes Partnership (OMOP) Common Data Model, which aims at harmonizing electronic medical records to facilitate participation on international distributed research.

The cohort includes all users of the Public Health Service of Navarra who, as of January 1st, 2012, had active codes of type 1 or type 2 diabetes (T89 and T90 of the International Classification of Primary Care, version 2, ICPC-2, respectively) in the Primary Care Electronic Medical Record System of Navarra (ATENEA) records. Patients with descriptions of diabetes different from T1D or T2D were

For peer review only - http://bmjopen.bmj.com/site/about/guidelines.xhtml

excluded, as well as when severe inconsistencies in the dates of diagnosis, birth, or death were found. Patients were also excluded if no registry of contact with the public health system was found either before the inclusion date and/or in the follow-up period. No other exclusion criteria were applied, and patients of all ages and conditions were considered, including T2D patients with onset during childhood and T1D patients with late onset during adulthood. Causes of early termination of the patient data extraction were death or change of community/country.

Figure 1 shows the flowchart of the creation of the cohorts. The classification of patients into T1D or T2D took into account that the validity of the T2D diagnosis had been assessed in a previous study,[9] but not that of the T1D diagnosis. Hence, the ICPC-2 codes and the descriptive field that goes with the code were first used, and after that, the classification procedure was complemented with the regional registry of T1D diabetes, which was legally approved by formal order 37/2014, on April 16th,[10] that includes all T1D patients with an onset date after 1989. More precisely, we first included all patients with ICPC-2 code T89 and T90 with active Individual Health Card in ATENEA. Second, we excluded patients with incompatible or incongruent information, such as to have died before 2012, to have unrealistic birth or onset dates, or to have diagnostic literals 'gestational diabetes' or 'pancreoprivic diabetes'. Third, using the regional registry of T1D diabetes, we maintained in the T2D cohort only patients that were not in the T1D registry, and passed all patients with T2D code to the T1D cohort if they were included in the T1D registry. Fourth, we excluded from the T1D cohort all patients that had onset date >1990 but were not in the T1D registry, and also all patients with onset date <1990 that were not treated with insulin. Finally, we excluded patients that had no contact with the health system before 2012, and patients that had no contact with the health system from 2012 to 2016. Combining the information from the health electronic records and administrative population datasets, two cohorts of prevalent patients with diabetes were created: the T1D CARDIANA cohort, with 1067 patients, and the T2D CARDIANA cohort, with 33842 patients.

For peer review only - http://bmjopen.bmj.com/site/about/guidelines.xhtml

During the follow-up of the first wave, 33 (3.1%) T1D patients and 455 (1.3%) T2D patients were lost to follow-up because of having moved to another region, and information for these patients was censored at this date accordingly.

The actual follow-up period of the cohort is five years, from January 1st 2012, to December 31st, 2016. The next data extraction process that will update longitudinal data and principal cardiovascular events is being conducted in late 2022 and early 2023, covering the period from January 1st 2017, to December 31st, 2021, and further extractions are planned in 5 year waves.

Variables, databases, and integration process

Sociodemographic and clinical variables of the defined CARDIANA T1D and CARDIANA T2D cohorts came from eight clinical and administrative databases: ATENEA, LAKORA-TIS, LAMIA, HCI, HIS-LEIRE (including the Minimum Basic Data Set at hospital discharge - MBDS), the population registry, the mortality registry, and the Type 1 diabetes registry. For future updates of the cohort, the Results Analysis Database of Navarra (BARDENA) will be used. A brief description of the original databases, which were extensively described elsewere,[11] is given in Supplementary Table S1, whereas a summary of the variables considered is given in Supplementary Table S2. In all, the information collected consists of all relevant structured data available from these sources generated during each contact of the patient with the health system. One set of variables was collected once and was considered fixed during the follow-up. These include the date of entry and/or exit from the health system, demographic and socioeconomic data such as the study level, lifestyle information such as tobacco use or physical activity level, the basic health zone the patients belongs to, coinsurance status[12], baseline comorbidities and past history of cardiovascular history using the ICPC-2 codification system, among others. Some new variables were created from these previous variables, such as the Charlson weighted score[13] or the GMA[14,15] comorbidity score. The other set of variables

For peer review only - http://bmjopen.bmj.com/site/about/guidelines.xhtml

was collected longitudinally using a time-dependent structure and included all analytic results that occurred during follow-up as well as pharmacologic treatments, health service use and fatal and nonfatal cardiovascular events. For these time-dependent variables, the date on which they occurred was also included. Cardiovascular events were considered to occur during the follow-up when CVD diagnostic codes were recorded in the mortality or the MBDS dataset, as defined in the recent SCORE2 study[16] (see the list of codes considered for fatal and non-fatal CVD in Supplementary Table S3).

The integration procedure was conducted by the Statistic Institute of Navarra (NASTAT) and the Directorate-General for Informatics, Telecommunications, and Innovation of the Health Department of Navarra, who supervised the data extraction and guaranteed fulfilment of the law in terms of personal data protection. Afterward, the anonymized databases were provided to the research team.

Patient and public involvement

None.

Findings to date

The T1D and T2D CARDIANA cohorts consisted of 1067 and 33842 patients, respectively. Their sociodemographic characteristics are given in Table 1. No adjustment has been included due to the descriptive nature of the objective, but information on both cohorts is presented in parallel. Patients in both cohorts were primarily men (57.4% and 55.7% in T1D and T2D cohorts, respectively), and only 5% were immigrants. Compared to patients in the T2D cohort, patients in the T1D cohort were much younger (mean age 36.9 years in T1D vs. 69.4 years in T2D), had a higher probability of being part of the workforce (84.5% vs. 26.6%), had a higher income level (38.3% vs. 27.5% had over 18,000 € per year) and also higher educational attainment (17.8% vs. 4.7% had university studies).

For peer review only - http://bmjopen.bmj.com/site/about/guidelines.xhtml

Health related patients' status at baseline, including lifestyle data, laboratory tests values, and office measured parameters and comorbidities are given in Table 2. The mean duration of diabetes was three years higher in T1D patients than in T2D patients (11.0 vs. 8.1 years), but their comorbidity indices were lower, with a weighted Charlson score equal to 1.2 vs. 2.1 and a weighted GMA equal to 6.0 vs. 11.4, respectively. Similarly, T1D patients have much lower prevalence of cardiovascular disease history (4.9% vs. 23.8%), were more frequently active (71.5% vs 55.9%) and alcohol abstinent (69.0% vs 66.5%) but had higher probability of being smokers (32.2% vs 17.7%).

Regarding clinical and laboratory test parameters, T1D patients showed much better control of their body mass index (25.7 vs. 30.6 kg/m^2) but worse glycemic control of their HbA1c levels (8.2% vs. 7.1%). They also had better control of the other metabolic parameters considered, namely, high-density lipoprotein (62.0 vs. 48.9 mg/dL), low-density lipoprotein (105.7 vs. 111.4 mg/dL), triglycerides (84.2 vs. 142.7 mg/dL) and albumin to creatinine ratio levels (13.4 vs. 35.9).

The use of primary care services during the last year before baseline (Table 3) was lower in T1D than in T2D patients for the total number of visits (17.1 vs. 21.1 visits/year) and for all types of visits, except for emergency visits (0.6 vs. 0.5 visits/year) and remote visits to nursing (4.1 vs. 1.1 visits/year). Active prescriptions of antihypertensive medication were 41 points less frequent in patients with T1D (15.2% vs. 56.2%), and their lipid-lowering treatment use was less than half (18.8% vs. 49.3%). Similar differences were observed in antithrombotic treatment prescription (15.6% vs 38.2%), but baseline glucose-lowering treatment prescription was higher in patients with T1D (83.6% vs. 67.2%).

Only 9 patients (0.8%; 95% CI [0.4, 1.6]) in the T1D cohort developed a CVD event, five of which were fatal (0.5% of the total cohort). In the same follow up period, 22 patients died from non-cardiovascular related events. For the T2D cohort, 2602 (7.7%; 95% CI [7.4, 8.0]) had an event and

For peer review only - http://bmjopen.bmj.com/site/about/guidelines.xhtml

1268 of them were fatal (3.7% of the total cohort). During this follow up, 5072 patients died from non-cardiovascular related events.

For the T2D cohort, the occurrence of CVD events along the follow-up has been associated with physical activity, with estimated ORs after matching and adjusting for confounders equal to 0.84 (95% CI: 0.66-1.07) for the partially active group and 0.71 (95% CI: 0.56-0.91) for the active group, compared with patients in the non-active group.[17] Note that, in this study, a slightly different CVD outcome was considered.

Strengths and limitations

The main strength of this study is that the CARDIANA cohorts integrate exhaustive clinical, socioeconomic and behavioral information from all available administrative and clinical data sources, providing a complete framework to assess the course of the disease in patients with diabetes and the factors that affect it. Especially in relation to socioeconomic variables, these cohorts have individual information on country of origin, working status, educational level and income, which are not frequently available, with most studies using area-level proxies. Another strength is that data have been subjected to quality control procedures before and after database integration.

The main limitation of this study is the possible presence of bias resulting from the use of existing electronic clinical records, which may affect different methodological aspects. First, T2D patients without a diabetes code in the ATENEA records were not included. Although the validity of the code has been satisfactory asssessed,[9] undiagnosed patients have not been included, which in Spain it has been estimated that could account for 4-6% of the overall prevalence of 11-14%.[18,19] Second, data completeness can be low for some variables dependent on physicians' idiosyncratic reporting procedures, such as tobacco use or physical activity, and some variables that have been considered

For peer review only - http://bmjopen.bmj.com/site/about/guidelines.xhtml

fixed may have changed along the follow-up. Although an effort has been made to complement variables with others that had a text format, information bias may be present, so imputation methods and sensitivity analyses will be required. Third, electronic prescriptions were only fully implemented in 2014, and at baseline it is estimated that 8-10% of the total prescriptions have not been accounted for. Fourth, patients without any contact with the regional public health system because of using exclusively private health institutions were not included. Nevertheless, it is estimated that these patients account for less than 1% in the region.[8]

Collaboration

Requests for collaborative studies are welcome, upon request with a description of the planned projects from berta.ibanez.beroiz@navarra.es. They will only be considered after the approval of the research ethics committee from the solicitor institution and from the Navarra health system – Osasunbidea and the NASTAT institutions – responsible for the clinical information and the population information.

*** ***

Ethics approval

The study protocol was approved by the Ethics Committee of Clinical Research of Navarra (Project 2015/111). This Committee approved, on August 19th 2022, to update the time-window of the cohorts to December 31st, 2021. This study has a retrospective nature, and data were irreversibly anonymized prior to transfer to the research team. The study was conducted according to the amended Declaration of Helsinki, Organic Law 3/2018, the General Data Protection Regulation (EU) 2016/679 and International Guidelines for Ethical Review of Epidemiological Studies.

For peer review only - http://bmjopen.bmj.com/site/about/guidelines.xhtml

Contributors

BI and KC designed the study, researched the data and reviewed the manuscript. IT analyzed the data and wrote the manuscript. JLi, AG and ME researched the data and reviewed the manuscript. LF and MJG participated in the design of the study, in the acquisition of the T1D registry data and reviewed the manuscript. OL, JG, and AO participated in the acquisition and validation of the ATENEA, LAKORA, LAMIA, HIS-Leire and HCI datasets and reviewed the manuscript. LA participated in the acquisition and validation of the population registry and reviewed the manuscript. CM participated in the acquisition and validation of the mortality registry database and reviewed the manuscript. JLa participated in the design of the study, contributed to the interpretation of the results and reviewed the manuscript. All authors have approved this version of the manuscript.

Funding

This work has received funding from the Instituto de Salud Carlos III through grant PI15/02196 and via the CONCEPT project (grants PI19/00154, PI19/00381). It has also received funding from REDISSEC RD16/0001/0014 and RICAPPS RD21/0016/0016, two Spanish Networks supported by Carlos III Health Institute and the European Regional Development Funding (FEDER). All funds are public and had no role in the design of the study, analysis or writing of the paper.

Data availability statement

Proposals for collaborative studies to share information on data are welcome, upon request with a description of the planned projects from berta.ibanez.beroiz@navarra.es. They will only be considered after the approval of the research ethics committee from the solicitor institution and from the Navarra health system – Osasunbidea and the NASTAT institutions – responsible for the clinical information and the population information.

For peer review only - http://bmjopen.bmj.com/site/about/guidelines.xhtml

For peer review only

Acknowledgements

The authors thank all professionals who participated in the acquisition of the data, especially Javier Baquedano, Adriana Rivero, Mª Ángeles Nuin and Alberto Jiménez-Idiazábal. The authors belong to the group RD16/0001/0014 of REDISSEC and to the group RD21/0016/0016 of RICAPPS.

Competing interests

None declared.

For peer review only

References

1. International Diabetes Federation. IDF Diabetes Atlas, 10th edition. 2022. Available from: https://www.diabetesatlas.org

2. The Lancet. Turning evidence into action on diabetes. *Lancet* [Internet]. Elsevier Ltd; 2020;**396**(10262):1535. Available from: http://dx.doi.org/10.1016/S0140-6736(20)32412-0

3. Peters SAE, Huxley RR, Sattar N, Woodward M. Sex Differences in the Excess Risk of Cardiovascular Diseases Associated with Type 2 Diabetes: Potential Explanations and Clinical Implications. *Curr Cardiovasc Risk Rep*. 2015;**9**(7):1–7.

4. Livingstone SJ, Looker HC, Hothersall EJ, et al. Risk of Cardiovascular Disease and Total Mortality in Adults with Type 1 Diabetes: Scottish Registry Linkage Study. *PLoS Med*. 2012;**9**(10).

5. Forbes JM, Cooper ME. Mechanisms of diabetic complications. *Physiol Rev*. 2013;**93**(1):137–188.

6. Galbete A, Tamayo I, Librero J, Enguita-Germán M, Cambra K, Ibáñez-Beroiz B. Cardiovascular risk in patients with type 2 diabetes: A systematic review of prediction

For peer review only - http://bmjopen.bmj.com/site/about/guidelines.xhtml

models. *Diabetes Res Clin Pract*. 2022;**184**.

7. Ibáñez B, Galbete A, Goñi MJ, et al. Socioeconomic inequalities in cardiometabolic control in patients with type 2 diabetes. *BMC Public Health*. BMC Public Health; 2018;**18**(1):1–9.

8. Asistencia sanitaria. Cifras relativas. Instituto Nacional Estadística (INE), 2018. https://www.ine.es/jaxi/Tabla.htm?tpx=47935&L=0.

9. Moreno-Iribas C, Sayon-Orea C, Delfrade J, et al. Validity of type 2 diabetes diagnosis in a population-based electronic health record database. *BMC Med Inform Decis Mak*. BMC Medical Informatics and Decision Making; 2017;**17**(1):1–6.

10. Orden Foral 37/2014. Navarra,Spain: https://bon.navarra.es/es/anuncio/-/texto/2014/88/10; 2014.

11. Moulis G, Ibañez B, Palmaro A, et al. Cross-national health care database utilization between spain and france: results from the epichronic study assessing the prevalence of type 2 diabetes mellitus. *Clin Epidemiol*. 2018;**10**:863–874.

12. Pozo-Rubio R del, Pardo-García I, Escribano-Sotos F. El copago de dependencia en España a partir de la reforma estructural de 2012. *Gac Sanit*. 2017;**31**(1):23–29.

13. Charlson ME, Pompei P, Ales KL, MacKenzie CR. A new method of classifying prognostic comorbidity in longitudinal studies: development and validation. *J Chronic Dis*. England; 1987;**40**(5):373–383.

14. González González AIG, Miquel Gómez AM, Rodríguez Morales D, et al. Concordancia y utilidad de un sistema de estratificación para la toma de decisiones clínicas. *Atención Primaria*. 2017;**49**(4):240–247.

For peer review only - http://bmjopen.bmj.com/site/about/guidelines.xhtml

For peer review only

15. Monterde D, Vela E, Clèries M. Adjusted morbidity groups: A new multiple morbidity measurement of use in Primary Care. *Aten Primaria* [Internet]. Elsevier España, S.L.U.; 2016;**48**(10):674–682. Available from: http://dx.doi.org/10.1016/j.aprim.2016.06.003

16. SCORE2 working group and ESC Cardiovascular risk collaboration, SCORE2 risk prediction algorithms: new models to estimate 10-year risk of cardiovascular disease in Europe, *European Heart Journal*, Volume 42, Issue 25, 1 July 2021, Pages 2439–2454.

17. Enguita-Germán M, Tamayo I, Galbete A, Librero J, Cambra K, Ibáñez-Beroiz B. Effect of physical activity on cardiovascular event risk in a population-based cohort of patients with type 2 diabetes. *Int J Environ Res Public Health*. 2021;**18**(23).

18. Soriguer F, Goday A, Bosch-Comas A, et al. Prevalence of diabetes mellitus and impaired glucose regulation in Spain: The Di@bet.es Study. *Diabetologia*. 2012;**55**(1):88–93.

19. Aguayo A, Urrutia I, González-Frutos T, et al. Prevalence of diabetes mellitus and impaired glucose metabolism in the adult population of the Basque Country, Spain. *Diabet Med*. 2017;**34**(5):662–666.

FIGURE TITLE:

Figure 1: Flowchart for creation of the type 1 and type 2 prevalent diabetes cohorts

For peer review only

Table 1: Demographic and socioeconomic characteristics of the T1D and T2DCARDIANA cohorts at baseline (January 1st, 2012)

	T1D CARDIANA Cohort			T2D CARDIANA Cohort		
	Male	Female	**Total**	Male	Female	**Total**
**n**	612	455	1067	18840	15002	33842
**Age**, Mean (SD)	36.6 (15.9)	37.2 (17.8)	36.9 (16.7)	67.1 (12.3)	72.3 (13.0)	69.4 (12.8)
**Working status, n(%)**						
Unemployed	36 (7.8)	21 (6.7)	57 (7.3)	836 (5.0)	609 (5.8)	1445 (5.3)
Working	392 (84.5)	264 (84.6)	656 (84.5)	5506 (33.1)	1713 (16.3)	7219 (26.6)
Pensioner	36 (7.8)	27 (8.7)	63 (8.1)	10308 (61.9)	8157 (77.8)	18465 (68.1)
(Missing)	*148*	*143*	*291*	*2190*	*4523*	*6713*
**Continent of origin, n(%)**						
Spain	559 (94.6)	415 (94.7)	974 (94.7)	17645 (95.9)	13853 (94.7)	31498 (95.3)
Europe	15 (2.5)	5 (1.1)	20 (1.9)	293 (1.6)	240 (1.6)	533 (1.6)
Africa	9 (1.5)	6 (1.4)	15 (1.5)	171 (0.9)	115 (0.8)	286 (0.9)
America	8 (1.4)	11 (2.5)	19 (1.8)	269 (1.5)	399 (2.7)	668 (2.0)
Asia	0 (0.0)	1 (0.2)	1 (0.1)	23 (0.1)	29 (0.2)	52 (0.2)
Australia	0 (0.0)	0 (0.0)	0 (0.0)	1 (0.0)	0 (0.0)	1 (0.0)
(Missing)	*21*	*17*	*38*	*438*	*366*	*804*
**Copayment category, n(%)**						
<18000	338 (56.4)	301 (68.9)	639 (61.7)	11445 (62.9)	12223 (84.6)	23668 (72.5)
≥18000	261 (43.6)	136 (31.1)	397 (38.3)	6747 (37.1)	2229 (15.4)	8976 (27.5)
(Missing)	13	18	31	648	550	1198
**Study level, n(%)**						
No formal education	132 (22.8)	79 (18.5)	211 (21.0)	5245 (28.6)	6033 (41.4)	11278 (34.3)
Primary School	226 (39.1)	157 (36.9)	383 (38.1)	9744 (53.1)	7410 (50.8)	17154 (52.1)
High School	136 (23.5)	95 (22.3)	231 (23.0)	2227 (12.1)	711 (4.9)	2938 (8.9)
University level	84 (14.5)	95 (22.3)	179 (17.8)	1121 (6.1)	424 (2.9)	1545 (4.7)
(Missing)	*34*	*29*	*63*	*503*	*424*	*927*
**Mean income, Mean (SD)**	12011.6 (1803.8)	12099.3 (1742.3)	12048.9 (1777.6)	11748.2 (1845.8)	11531.6 (1739.5)	11652.3 (1802.7)
**Income Quintile, n(%)**						
[7300,10565)	128 (21.7)	78 (17.8)	206 (20.0)	3539 (19.2)	3144 (21.5)	6683 (20.2)
[10565,11416)	110 (18.6)	97 (22.1)	207 (20.1)	3515 (19.1)	3099 (21.2)	6614 (20.0)
[11416,12240)	122 (20.6)	84 (19.2)	206 (20.0)	3577 (19.4)	2980 (20.4)	6557 (19.8)
[12240,13394)	118 (20.0)	88 (20.1)	206 (20.0)	3820 (20.8)	2799 (19.1)	6619 (20.0)
[13394,17708]	113 (19.1)	91 (20.8)	204 (19.8)	3949 (21.5)	2615 (17.9)	6564 (19.9)
(Missing)	*21*	*17*	*38*	*440*	*365*	*805*

Percentage for each category are column percentages (% of patients in each category for each cohort), unless otherwise indicated (mean and standard deviations- SD are given in quantitative variables)

For peer review only - http://bmjopen.bmj.com/site/about/guidelines.xhtml

Table 2: Clinical and lifestyle characteristics of the T1D and T2D CARDIANA cohorts at baseline (January 1st, 2012)

		T1D CARDIANA Cohort			T2D CARDIANA Cohort		
		Male	Female	**Total**	Male	Female	**Total**
**n**		612	455	1067	18840	15002	33842
Clinical parameters, mean (SD)							
Duration of diabetes (years)		10.8 (9.2)	11.1 (9.1)	11.0 (9.1)	7.8 (5.8)	8.5 (6.3)	8.1 (6.0)
Body Mass Index (Kg/m2)		26.2 (4.5)	25.2 (5.0)	25.7 (4.7)	30.2 (5.3)	31.0 (6.4)	30.6 (5.8)
Systolic Blood Pressure (mm Hg)		124.0(19.0)	121.4(19.9)	122.9(19.4)	135.5 (16.9)	135.7 (17.9)	135.6 (17.3)
Diastolic Blood Pressure (mm Hg)		72.3 (12.3)	71.0 (9.8)	71.7 (11.2)	76.6 (10.5)	75.8 (10.5)	76.2 (10.5)
Laboratory tests, mean (SD)							
HbA1c (%)		8.2 (1.6)	8.2 (1.4)	8.2 (1.5)	7.0 (1.3)	7.1 (1.3)	7.1 (1.3)
Fasting glucose (mg/dL)		179.9(97.6)	172(80.5)	176.3(89.3)	141.6 (44.6)	139.0 (45.4)	140.4 (45.0)
Total Cholesterol (mg/dL)		188(42.1)	188(31.2)	188(36.9)	183.2 (39.2)	194.0 (37.9)	188.0 (39.0)
High Density Lipoprotein (mg/dL)		57.5 (17.0)	68.4 (17.8)	62.0 (18.2)	46.3 (13.3)	52.1 (14.4)	48.9 (14.1)
Low Density Lipoprotein (mg/dL)		107.2(29.8)	103.6(28.7)	105.7(29.4)	108.9 (31.9)	114.4 (32.4)	111.4 (32.2)
Triglicerides (mg/dL)		90.7 (58.5)	75.1 (37.7)	84.2 (51.4)	141.5 (77.3)	144.1 (68.9)	142.7 (73.7)
Creatinine (mg/dL)		0.9 (0.3)	0.7 (0.2)	0.8 (0.3)	1.1 (0.6)	0.9 (0.5)	1.0 (0.6)
Albumin to creatinine ratio		12.9 (30.9)	14.0 (31.5)	13.4 (31.2)	40.2 (165.6)	30.4 (134.0)	35.9 (152.4)
Lifestyle data, n(%)							
Smoking Status	Non Smoker	149 (49.2)	150 (60.2)	299 (54.2)	6009 (37.9)	11025(85.1)	17034(59.1)
	Ex-smoker	51 (16.8)	24 (9.6)	75 (13.6)	5810 (36.6)	887 (6.8)	6697 (23.2)
	Smoker	103 (34.0)	75 (30.1)	178 (32.2)	4052 (25.5)	1041 (8.0)	5093 (17.7)
	(Missing)	*309*	*206*	*515*	*2969*	*2049*	*5018*
Alcohol	Abstinent	150 (59.5)	159 (81.1)	309 (69.0)	7082 (47.5)	10805(90.1)	17887(66.5)
	Moderate drinker	95 (37.7)	37 (18.9)	132 (29.5)	7044 (47.3)	1137 (9.5)	8181 (30.4)
	Heavy drinker	7 (2.8)	0 (0.0)	7 (1.6)	777 (5.2)	44 (0.4)	821 (3.1)
	(Missing)	*360*	*259*	*619*	*3937*	*3016*	*6953*
Physical activity	Inactive	7 (4.0)	11 (7.6)	18 (5.6)	1431 (9.8)	1931 (16.1)	3362 (12.6)
	Partially active	38 (21.7)	35 (24.3)	73 (22.9)	3966 (27.2)	4405 (36.8)	8371 (31.5)
	Active	130 (74.3)	98 (68.1)	228 (71.5)	9207 (63.0)	5647(47.1)	14854(55.9)
	(Missing)	*437*	*311*	*748*	*4236*	*3019*	*7255*
Comorbidities							
Charlson score, mean (SD)		1.2 (0.8)	1.2 (0.8)	1.2 (0.8)	2.2 (1.7)	2.1 (1.6)	2.1 (1.7)
GMA[a] score, mean (SD)		5.5 (3.8)	6.7 (4.7)	6.0 (4.2)	10.7 (5.8)	12.2 (5.9)	11.4 (5.9)
Previous CVD		31 (5.1)	21 (4.6)	52 (4.9)	4804 (25.5)	3236 (21.6)	8040 (23.8)
Diabetes related comorbidities, n(%)							
Retinopathy (%)	Yes	25 (35.2)	11 (18.6)	36 (27.7)	670 (15.3)	616 (18.9)	1286 (16.8)
	(Missing)	*541*	*396*	*937*	*14451*	*11738*	*26189*
Amputation	Yes	1 (1.2)	0 (0.0)	1 (0.7)	160 (2.0)	54 (0.8)	214 (1.5)
	(Missing)	*532*	*390*	*922*	*10865*	*8252*	*19117*
Diabetic foot risk	None	4 (5.9)	4 (6.2)	8 (6.0)	230 (3.2)	212 (3.5)	442 (3.3)
	Superficial ulcer	53 (77.9)	56 (86.2)	109 (82.0)	6048 (83.6)	4809 (79.7)	10857(81.8)
	Deep tissue ulcers w/o abcess	8 (11.8)	3 (4.6)	11 (8.3)	652 (9.0)	704 (11.7)	1356 (10.2)
	Deep tissue ulcers w abcess	2 (2.9)	1 (1.5)	3 (2.3)	182 (2.5)	244 (4.0)	426 (3.2)
	Localized gangrene	1 (1.5)	1 (1.5)	2 (1.5)	113 (1.6)	62 (1.0)	175 (1.3)
	Extensive gangrene	0 (0.0)	0 (0.0)	0 (0.0)	10 (0.1)	4 (0.1)	14 (0.1)
	(Missing)	*544*	*390*	*934*	*11605*	*8967*	*20572*

Percentage for each category are column percentages (% of patients in each category for each cohort) unless otherwise indicated (mean and standard deviations- SD are given in quantitative variables)
[a]GMA: Adjusted Morbidity groups

For peer review only - http://bmjopen.bmj.com/site/about/guidelines.xhtml

Table 3: Use of Primary Care Services during the year previous to baseline (2011) and active prescriptions at baseline (January 1st, 2012)

	T1D CARDIANA Cohort			T2D CARDIANA Cohort		
	Male	Female	**Total**	Male	Female	**Total**
**n**	612	455	1067	18840	15002	33842
**Total visits,** mean (SD)	15.9 (11.4)	19.0 (12.6)	17.1 (12.2)	19.4 (19.1)	23.2 (20.8)	21.1 (19.9)
**Visits at Office, by professional,** mean(SD)						
Nursing	5.2 (6.7)	5.6 (6.9)	5.4 (6.8)	7.1 (10.3)	7.6 (9.7)	7.3 (10.1)
Physician	3.8 (4.3)	5.2 (5.5)	4.4 (4.9)	6.4 (5.8)	7.3 (6.5)	6.8 (6.1)
Social Worker	0.8 (1.4)	1.3 (3.4)	1.0 (2.5)	1.7 (2.4)	1.9 (3.6)	1.7 (3.0)
Emergency	0.1 (0.3)	0.1 (0.5)	0.1 (0.4)	0.1 (0.6)	0.2 (0.9)	0.1 (0.8)
Other	0.6 (1.7)	0.7 (1.5)	0.6 (1.6)	0.5 (2.7)	0.6 (1.8)	0.5 (2.3)
**Visits at home, by professional,** mean(SD)						
Nursing	0.1 (0.8)	0.0 (0.4)	0.1 (0.7)	0.7 (5.7)	1.4 (7.8)	1.0 (6.7)
Physician	0.1 (0.3)	0.0 (0.2)	0.0 (0.3)	0.3 (1.7)	0.6 (2.2)	0.4 (1.9)
Social Worker	0.0 (0.1)	0.0 (0.2)	0.0 (0.2)	0.1 (1.2)	0.2 (1.6)	0.1 (1.4)
Emergency	0.0 (0.0)	0.0 (0.1)	0.0 (0.0)	0.0 (0.1)	0.0 (0.2)	0.0 (0.2)
Other	0.0 (0.3)	0.0 (0.1)	0.0 (0.3)	0.1 (0.6)	0.1 (0.8)	0.1 (0.7)
**Remote visits, by professional,** mean(SD)						
Nursing	3.9 (4.9)	4.3 (5.6)	4.1 (5.2)	0.9 (2.3)	1.3 (2.8)	1.1 (2.6)
Physician	1.4 (2.3)	1.8 (2.9)	1.5 (2.6)	1.5 (3.1)	2.0 (3.9)	1.7 (3.5)
Social Worker	0.0 (0.1)	0.0 (0.1)	0.0 (0.1)	0.0 (0.2)	0.0 (0.2)	0.0 (0.2)
Emergency	0.0 (0.2)	0.0 (0.2)	0.0 (0.2)	0.0 (0.4)	0.1 (0.6)	0.1 (0.5)
**Drug treatments use,** n(%)						
Antihypertensive	95 (15.5)	67 (14.7)	162 (15.2)	10303(54.7)	8769(58.5)	19072(56.4)
Glucose-lowering	519 (84.8)	373 (82.0)	892 (83.6)	12630(67.0)	10099(67.3)	22729(67.2)
Lipid-lowering	122 (19.9)	79 (17.4)	201 (18.8)	9423(50.0)	7249(48.3)	16672(49.3)
Antithrombotic	67 (10.9)	47 (10.3)	114 (10.7)	7722(41.0)	5262(35.1)	12984(38.4)

Percentage for each category are row percentages (% of patients with each treatment in each cohort), unless otherwise indicated (mean and standard deviations- SD are given in quantitative variables)

For peer review only - http://bmjopen.bmj.com/site/about/guidelines.xhtml

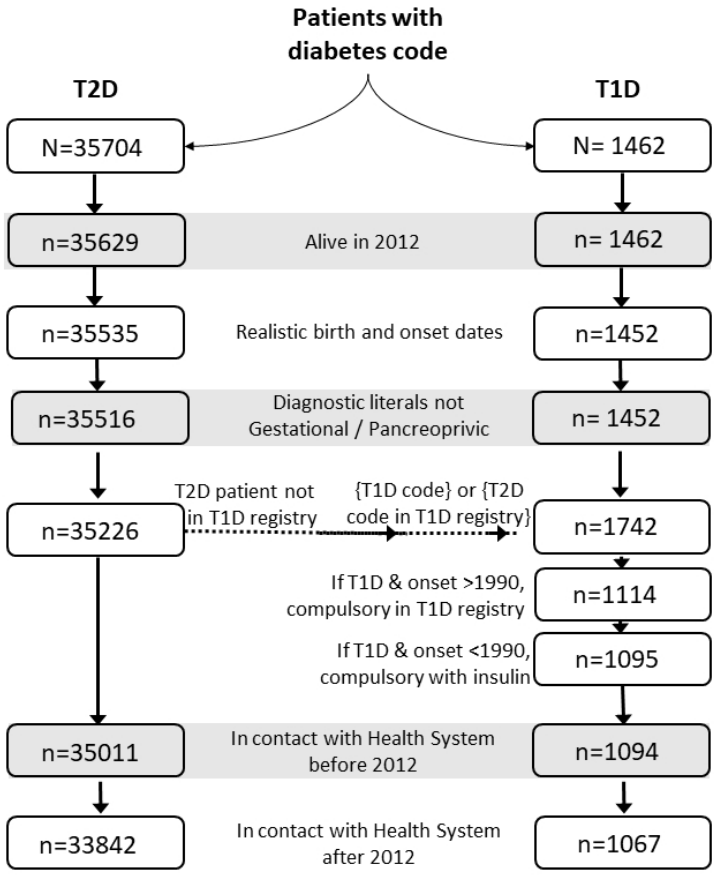

Figure 1: Type 1 and type 2 diabetes prevalent cohort creation flowchart

190x275mm (96 x 96 DPI)

Supplementary Table S1: Original Data Sources used for the creation of the T1D and T2D cohorts[Ω]

ATENEA –The Primary Care Electronic Medical Record System of Navarra
What contains: out-hospital data. Includes demographic information, visits to primary care services (data and type), health problems, life style, detailed clinical data, laboratory results and drug prescription data.<br>*Code system used:* ICPC-2 International Classification of Primary Care, version 2<br>*Implemented:* in 2003, with a 100% of use in 2008
LAKORA – Population Information System (within the Health system)
What contains: Basic information on coverage, insurance modality, pharmaceutical copayment status, primary healthcare district, country of origin and other administrative data.<br>*Code system used*: free text<br>*Implemented: in 2003,* with a 100% of use in 2008
MORTALITY Registry
What contains: Date and cause of death<br>*Code system used*: International Classification of Diseases ICD-10-ES
HIS-LEIRE (with the Minimum Basic Data Set, MBDS)
What contains: It contains the MBDS, with socio-demographic and clinical information on all hospital discharges and major ambulatory surgery in the hospitals of Navarra, including partnership hospitals. It also contains information about visits to specialist or to day hospitals<br>*Code system used*: International Classification of Diseases; ICD9CM until December, 2015 and ICD-10-ES from January, 2016 on.<br>*Implemented*: The MBDS was compulsory in Spain since 1992. HIS-LEIRE had 100% of use in 2003
HCI- Clinical data from specialized care
What contains: comprehensive information of specialized care, outpatient consultation, lab test, images, visits and other data from specialized care.<br>*Code system used*: free text<br>*Implemented*: In 2001, with 100% of use in 2008
T1D registry
What contains: demographic and clinical information of all patients with T1D diabetes to estimate incidence, prevalence, morbidity and mortality of T1D patients<br>*Implemented*: in 2014, legally approved by formal order 37/2014
Population registry
What contains: At individual level: study level, nationality, labor force status (employed, unemployed, pensioner); at area-level (census tract): average income (2013), % unemployed, %immigration, % people without studies<br>*Code system used*: For study level: Normalized Classification of Education (CNE) used for census in the INE (National Institute of Statistics)[£]
LAMIA
What contains: e-prescription paper-free system with both prescriptions and dispensations, connected to all community pharmacies in the region<br>*Code system used*: Anatomical Therapeutic Chemical (ATC) code<br>*Implemented*: In 2013, with 100% of use in 2014. Before 2014, information on prescriptions was obtained from ATENEA.

[Ω] For future updates of the cohort, the Results Analysis Database of Navarre (BARDENA) will be used

[£] https://idapadron.ine.es/repositorio/legislacion/Anexo%20II.pdf

For peer review only

Supplementary Table S2: Data information available in the T1D and T2D cohorts, by type of information and data source

Data source	Variables
**Socioeconomic data**	
Population Registry	Date of birth, country of origin, study level, marital status, working status and mean income of the assigned basic health zone
LAKORA-TIS	Pharmaceutical co-payment category
ATENEA-TIS	Date of birth, date of inclusion/withdrawal in the Navarra health system, general practitioner identification
**Previous diagnosis and past history of diseases**	
ATENEA	Weighted GMA, Past-history of myocardial infarction, congestive heart failure, cerebrovascular disease, peripheral vascular disease, retinopathy, neuropathy, nephropathy, foot lesions, dementia, chronic pulmonary disease, rheumatic disease, peptic ulcer disease, hemiplegia or paraplegia, neoplasm, liver disease, AIDS/HIV. Past history of CVD events coded in one of the following ICPC-2 codes: K74, K75, K76, K77, K89, K90, K91
**Personal cardiovascular risk factors, laboratory test and lifestyle behavior**	
ATENEA	Sex, age, physical activity, alcohol consumption, smoking status, self-management of pharmacological treatment, time since diagnosis, office, and laboratory test parameters (and dates): weight, height, systolic blood pressure (SBP), diastolic blood pressure (DBP), Total Cholesterol, LDL, HDL, triglycerides, creatinine, haemoglobin, glycated haemoglobin, fasting glucose, glomerular filtration rate (GFR), urine albumin to creatinine ratio.
The HCI database	Data collected by specialist care units: office and laboratory test parameters and procedures (date and value)
**Use of health services**	
HIS-LEIRE	Hospital or emergency room admissions, diagnostics, procedures and dates of admission and discharge. Number of consultations to specialist by medical specialty
ATENEA	Number of yearly telephonic/physical consultation, office/home-based consultation and type of service provided (nurse/ medical/social worker/Emergency room)
**Drug treatment use**	
LAMIA	Treatment ATC code, date of prescription and date of dispensation
ATENEA	Treatment ATC code and date of prescription (before 2014)
**Diabetes classification**	
T1D registry	Type 1 diabetes confirmation and onset date
**Outcomes**	
Mortality registry	Cause of death and date ICD-10-ES
HIS-LEIRE/MBDS	Minimum Basic Data Set (MBDS): ICD-9CM until December 31st, 2015 and ICD-10-ES from January 1st, 2016.

Supplementary Table S3: ICD-9 and ICD-10 codes for the definition of the outcome

**Fatal CVD event**		
Endpoints included	*ICD10-codes*	*ICD9-codes*
Hypertensive disease	I10-16	401 – 405
Ischemic heart disease	I20-25	410 - 414
Arrhythmias, heart failure	I46-52	426 - 429
Cerebrovascular disease	I60-69	430 - 438
Atherosclerosis/AAA	I70-73	440 - 443
Sudden death and death within 24h of symptom onset	R96.0-96.1	798.1 , 798.2
Excluded from the above endpoint:		
Myocarditis, unspecified	I51.4	426.7
Subarachnoid haemorrhage	I60	429
Subdural haemorrhage	I62	430
Cerebral aneurysm	I67.1	432.1
Cerebral arteritis	I68.2	437.3
Moyamoya	I67.5	437.4
Non-fatal CVD event		
Endpoints included		
Non-fatal myocardial infarction	I21-I23	410
Non-fatal stroke	I60-69	430-438
Excluded from the non-fatal stroke endpoint:		
Subarachnoid hemorrhage	I60	429
Subdural hemorrhage	I62	430
Cerebral aneurysm	I67.1	432.1
Cerebral arteritis	I68.2	437.3
Moyamoya	I67.5	437.4

Cardiovascular Disease (CVD) event defined with Codes considered in SCORE2 [16]
Only primary codes are considered

review only
